# Sexual lives and reproductive health outcomes among persons with disabilities: a mixed-methods study in two districts of Ghana

**DOI:** 10.1186/s12978-024-01810-4

**Published:** 2024-05-23

**Authors:** Abdul-Aziz Seidu, Bunmi S. Malau-Aduli, Kristin McBain-Rigg, Aduli E. O. Malau-Aduli, Theophilus I. Emeto

**Affiliations:** 1 Public Health & Tropical Medicine, College of Public Health, Medical and Veterinary Sciences, James Cook University, Townsville, QLD 4811 Australia; 2 Department of Population and Health, University of Cape Coast, P.O. Box UC 182, Cape Coast, Ghana; 3 College of Medicine and Dentistry, James Cook University, Townsville, QLD 4811 Australia; 4 School of Medicine and Public Health, University of Newcastle, Newcastle, NSW 2308 Australia; 5 School of Environmental and Life Sciences, The University of Newcastle, Callaghan, NSW 2308 Australia; 6 World Health Organization Collaborating Center for Vector-Borne and Neglected Tropical Diseases, James Cook University, Townsville, QLD 4811 Australia

**Keywords:** Disability, Ghana, Outcomes, Reproductive health, Sexual behaviour, Sexual health

## Abstract

**Introduction:**

People with disabilities (PwDs) constitute a large and diverse group within the global population, however, their sexual and reproductive health (SRH) needs are often neglected, particularly in low-and middle-income countries. This may result in adverse outcomes, such as sexually transmitted infections (STIs), unintended pregnancies, and experience of interpersonal violence (IV). This study aimed to assess the factors that influence the sexual lives of PwDs in two districts of Ghana.

**Methods:**

A sequential explanatory mixed-methods study design was used to collect data from PwDs. Quantitative data were obtained from 402 respondents using a pretested questionnaire, and qualitative data gathered from 37 participants using in-depth interviews. The quantitative data were analysed using descriptive and inferential statistics, while the qualitative data were analysed using inductive thematic analysis.

**Results:**

Most PwDs (91%) reported that they have ever had sex, which was associated with age, disability severity, and household size. The prevalence of poor SRH status, STIs, unintended pregnancy, pregnancy termination, and unsafe abortion were 10.5%, 5.7%, 6.4%, 21.6%, and 36.9% respectively. These outcomes were influenced by education, income, health insurance subscription, and proximity to a health facility. The prevalence of IV was 65%, which was related to disability type and severity. The qualitative data revealed five main themes: curiosity to engage in sexual activities, feelings of despair and insecurity with abled partners, preference for sexual relationships with other PwDs, IV and its perpetrators, and adverse SRH outcomes.

**Conclusion:**

The study findings indicate that most adult PwDs have ever had sex and they face various challenges in their sexual lives. They experience multiple forms of abuse and neglect from different perpetrators at different levels of society, which have negative impacts on their well-being. There is a need for comprehensive and inclusive interventions that address the SRH needs of PwDs, as well as the underlying social and structural factors that contribute to their vulnerability. Further research is recommended to explore the perspectives of stakeholders on how to improve the SRH outcomes of PwDs.

**Supplementary Information:**

The online version contains supplementary material available at 10.1186/s12978-024-01810-4.

## Plain Language summary

People with disabilities make up 16 percent of the world population, but their sexual and reproductive health (SRH) needs are often unmet, especially in poorer countries, contributing to adverse outcomes. This study assessed factors influencing the sex lives of PwDs in two districts of Ghana. Quantitative data were collected from 402 respondents, while qualitative data were collected from 37 participants. The results showed that age, severity of disability, and household size influence sexual activity. Many respondents also reported adverse SRH issues including STIs and unintended pregnancies. Sixty-five percent reported experiencing violence or abuse, which is related to their disability type and severity. The study also found that PwDs encounter many challenges in their sexual lives, including abuse and neglect. The findings suggest the need to pay attention to the sexual health needs and the social issues that make PwDs vulnerable to various abuses.

**Supplementary Information:**

The online version contains supplementary material available at 10.1186/s12978-024-01810-4.

## Introduction

The World Health Orgnanization (WHO) [1] reports that approximately one billion people, constituting 16% of the global population, live with some form of disability, with 80% of them residing in low- and middle-income countries (LMICs). In Africa, the disability prevalence is estimated at 10%, corresponding to 60–80 million individuals [2]. In Ghana, the disability prevalence is 8%, with the highest prevalence (17.3%) in the Ashanti region [3].

Sexual and reproductive health (SRH) is a fundamental human right and a vital aspect of public health [4]. According to the WHO, SRH is “a state of complete physical, emotional, mental, and social well-being in all matters relating to the reproductive system and to its functions and processes” [5]. Various international initiatives advocate for SRH recognition and promotion, including the International Conference on Population and Development–Cairo 1994 Program of Action [6], the Maputo Plan of Action [7], the Millennium Development Goals (MDG 5b) [8], and the sustainable development goals (SDG 3.7 and 5.6) [9, 10]. However, there remains a significant gap in accessing SRH information and services in sub-Sahara Africa, including Ghana [11], especially for persons with disabilities (PwDs) [1].

Sexual health, is expressed through an individual’s rights to freely express their sexuality within consensual relationships, safe sexual experiences, sexual consent, sexual autonomy, contraceptive use, marry, start a family, access comprehensive sexual information, and receive quality sexual healthcare [5]. As a fundamental human right, sexual health should be attainable and accessible to all including PwDs [5, 12], as it influences their overall quality of life and life satisfaction [13]. However, society often ignores or stigmatises the sexuality and sexual health of PwDs [14–16], assuming they are asexual or that their sexual well-being is less important [14–16]. Nevertheless, research shows that PwDs have similar sexual needs and desires as non-disabled people [13], and that they face higher risks of adverse SRH outcomes, such as sexual violence, unintended pregnancies, and sexually transmitted infections (STIs), including HIV and AIDS [17–22]. For instance, Parekh et al. [23] found that women with disabilities are more susceptible to STIs than those without disabilities. In Ghana, visually impaired young people often engage in sexual activity without sufficient knowledge about contraception or STIs [24]. Adverse SRH outcomes among PwDs are associated with multiple barriers at different levels of society: national-level, healthcare system/institutional, community-level, individual-level, and economic barriers [25–28].

While some literature exist on the SRH outcomes of PwDs [29–34], most of it is based on data from high-income countries such as United Kingdom [29, 31], the United States of America [32], and Germany [34].There is the need for more research on the sexual lives of PwDs in LMICs [12, 30, 33, 35–39], where they face more challenges and barriers to accessing SRH information and services [25–28]. Therefore, this study aimed to explore the sexual lives and reproductive health outcomes of PwDs in Ghana, a LMIC in sub-Saharan Africa. The study sought to answer the following research questions a) what are the sexual experiences and behaviour of PwDs? b) what are the SRH outcomes among PwDs in the Ashanti Region of Ghana? This study provides valuable and current evidence on sexual behaviour and experiences of PwDs in Ghana, to inform interventions to reduce the risk of adverse SRH outcomes and promote their sexual well-being.

### Theoretical framework

This paper is part of larger study adapting the health outcomes model [40, 41] as its theoretical framework. The study aims to assess the impact of health policies and interventions on the SRH outcomes among PwDs in Ghana. The model posits that health outcomes and behaviours are determined by the interaction of system characteristics, interventions, and client/population characteristics [42]. Client characteristics encompass socio-demographic (e.g. age, sex, marital status, level of education) and disability-related attributes (e.g. type of disability, disability severity), whereas interventions comprise strategies implemented by governmental entities, healthcare practitioners, and non-governmental organisations (NGOs) aimed at enhancing the SRH of PwDs. System characteristics pertain to the structure of healthcare systems and their responsiveness to health policies and interventions, as well as their interactions, which collectively influence individual SRH outcomes.

Within the model, outcomes encompass both positive and adverse SRH conditions. In the present study, outcomes consist of self-reported SRH issues among PwDs, including STIs, unintended and unwanted pregnancies, pregnancy termination, unsafe abortion, self-rated SRH, and experience of violence or abuse [42]. The model has been previously applied and explained in detail [28, 43]. The rationale for using this framework for the current study is that it captures the complex and multifaceted factors influencing the sexual experiences, behaviour, and reproductive health outcomes of PwDs.

## Methods

### Ethical considerations

Ethical clearance was obtained from three institutional review boards or committes: the Ghana Health Service (GHS) Ethics Review Committee (GHS-ERC: 005–0621), the Komfo Anokye Teaching Hospital (KATH) (KATH-IRB/RR/101/21), and the James Cook University (JCU) Human Ethics Committee (H8531). The study also received approval from the Regional Health Directorate in Kumasi and the Offinso North District Health Directorate in Akumadan. Additionally, the study obtained consent from the leaders of two disability groups (Ghana Association of the Blind and the Ghana Society of the Physically Disabled) in the Kumasi Metropolis and Offinso North District. Furthermore, the study ensured the confidentiality of the data. Prior to administering the study instrument, a document explaining the study objectives and confidentiality, and benefits of participation was provided to the participants. They were also informed that their involvement was voluntary and that they could withdraw from the study at any time devoid any repercussions. They were also assured that their data would be used only for academic purposes and that their identities would not be disclosed to anyone. Both written and verbal informed consents were obtained from all participants. The investigations were performed according to the Declaration of Helsinki.

### Study design

Mixed-methods designs are useful for disability and rehabilitation research, as they can capture the complexity and diversity of the experiences of PwDs [44]. This study employed a sequential explanatory mixed-methods design within a pragmatic paradigm [45, 46]. The first phase of this study involved conducting a survey among PwDs, while the second phase involved conducting in-depth interviews with participants who agreed to participate further. This design was chosen to enhance the depth and breadth of the study by combining the strengths of quantitative and qualitative data collection and analysis. By using both methods, a more holistic understanding of sexual lives, behaviour and reproductive health outcomes among PwDs could be achieved than by relying on either quantitative or qualitative data collection and analysis alone. Data for both phases was therefore triangulated and synthesised as shown in Table 4.

### Study area

Two different districts were selected as study areas to explore the possible variations in sexual lives and reproductive health outcomes among PwDs, the Kumasi Metropolis and the Offinso North District. The Kumasi Metropolis was chosen as an urban setting because it is the regional capital and has a high population density. The Offinso North District was chosen as a rural setting. The details of the study area have been previously described [28].

### Phase one: quantitative phase

#### Target population

This study included individuals with physical disabilities (e.g. Multiple Sclerosis, Muscular Dystrophy, Chronic Arthritis, Cerebral Palsy, Chronic Fatigue Syndrome, Fibromyalgia, Spina Bifida, Loss of limbs and Spinal Cord Injury) [47] and visual impairment (e.g. mild vision impairment or total blindness) [48]. According to the 2021 Population and Housing Census report, the Ashanti Region of Ghana has the highest proportion of PwDs (17.3%, 363,321). About 41.4% are males while 58.6% are females. The most prevalent types of disabilities in the region were visual/seeing impairments (4%) and physical impairments (3.6%). Participant inclusion criteria were: a) aged 18 or above (adulthood age in Ghana), b) having physical or visual impairment, and c) voluntary agreement to participate. Exclusion criteria were: a) under 18 years old, b) multiple disabilities, c) disabilities other than physical or visual impairment, and d) refusal to participate. More details regarding the study area and the inclusion and exclusion criteria can be found in previous studies [28, 43].

#### Sample size and sampling

The sample size for the quantitative phase of the study was calculated using the formula developed by Lwanga et al. [49], resulting in a calculated sample size of 402 PwDs. It is given as *n* = z^2^ *pq*/*d*^2^ where *n* = sample size, *p* = proportion of PwDs’ use of SRH services, d = level of uncertainty (5%/0.05), z^2^ = 95% level of confidence and *q* = 1 − *p*. The sampling frame was obtained from a list of PwDs provided by the leaders of the two disability groups. Participants were selected from this list using a systematic sampling technique [50] and contacted during regular meetings. More information regarding the sampling procedure can be found in a previous publication [28].

#### Survey instrument development and administration

The questionnaire items were developed from validated instruments and literature review [51, 52] and used to collect quantitative data from 402 PwDs. It consisted of multiple sections, but only three main sections were relevant for this study: client characteristics, sexual behaviour, and reproductive health outcomes (See Appendix 1). Trained research assistants (RAs), holding Master of Philosophy degrees in Population and Health and Disability and Rehabilitation degrees collected the data. To ensure that the questionnaire was fit for purpose, it was pre-tested among 30 PwDs before data collection. No modifications were made during the pretesting phase.

#### Measurement of variables

##### Outcome variables

The outcome variables were: ever engaged in sexual intercourse (yes=1, no=0), age at sexual initiation (18 or more, below 18 years), condom use at first sex(yes = 1, no = 0), HIV testing (yes = 1, no = 0), unintended pregnancy (yes = 1, no = 0), pregnancy termination (yes = 1, no = 0), self-reported STIs (yes = 1, no = 0), self-rated SRH (good = 1, bad = 0), sexual abuse (yes = 1, no = 0), emotional abuse(yes = 1, no = 0), physical abuse (yes = 1, no = 0), and interpersonal violence/abuse (IV)–experiencing at least one form of violence/abuse (yes = 1, no = 0). Evidence suggest that experience of violence is a continuum [53] hence presenting all the three key indicators of IV shows the clear picture of the magnitude of PwDs’ experience of the phenomenon. Details of these measures are in Appendix 1.

##### Independent variables

The independent variables included respondents’ demographic and disability-related characteristics. These were disability type (physically disabled, visually impaired), disability severity (mild, severe), age (18–29, 30–39, 40–49, 50–59, 60 and above), sex/gender (male, female), place of residence (Kumasi Metropolis, Offinso North), level of education (no formal education, primary, junior high school, senior high school/tertiary), religious affiliation (Christian, non-Christian), marital status (never married, married, separated/widowed/divorced), ethnicity (Akan, non-Akan), employment (not working, working), average monthly income (GHC0-99, GHC100-299, GHC300 +), National Health Insurance Scheme (NHIS) subscription (no, yes), household size (1–4, 5 +), and duration to the nearest health facility (less than 30 min, 30–59 min, 60 and above minutes). Details of these measures can be found in Appendix 1.

#### Quantitative data analyses

Data management and analysis were performed using Stata version 14 software (Stata Corporation, College Station, TX, USA). Descriptive statistics, such as frequencies and percentages, were used to analyse the survey data. Multivariable regression analysis, employing logit models was conducted to identify predictors of sexual behaviour and reproductive health outcomes. Complementary log–log models were employed for variables with skewed distribution (e.g. self-rated SRH status). Regression results were reported as adjusted odds ratios with their corresponding 95% confidence intervals (CIs). The choice of the reference group was informed by a priori and practical significance. Statistical significance was determined using a *p*-value threshold < 0.05. The quantitative phase of the study adhered to Strengthening the Reporting of Observational Studies in Epidemiology (STROBE) guidelines.

### Phase Two: Qualitative phase

#### Study design

The objective of the second phase of the study was to further explore the issues identified in the first phase and develop a more comprehensive understanding of the participants’ sexual behaviour, experiences and SRH outcomes. To accomplish this, a phenomenological study design within an interpretivist paradigm was utilised. Interpretivism, as a paradigm, posits that reality is subjective and may vary among different individuals [54]. As explained by Liamputtong [55] “phenomenology is a methodological approach with strong and dynamic foundation that seeks to understand, describe, and interpret human behaviour and the meaning individuals make of their experiences” (p. 117).

#### Participants and sampling

The participants for the qualitative phase of the study were selected from those who participated in the quantitative phase and expressed interest in follow-up interviews. A purposive selection technique was used to select 37 PwDs who represented different genders/ sexes, and types of disability. The selection of interview participants was also informed by the quantitative findings–to select participants whose issues warranted further exploration and participants’ demographic information, to ensure representation from all participant groups until data saturation was achieved. The inclusion criteria for the qualitative phase included: participants aged 18 or above, having physical or visual impairment and voluntary agreement to participate. Details can be found in a previous study [28].

#### Data collection instrument and procedure

The findings from the quantitative phase informed the development of interview questions for the qualitative phase. These findings guided adjustments to the in-depth interview guide for the qualitative phase, aimed at gaining deeper insights into the sexual behaviour, experiences and SRH outcomes of PwDs. For example, participants who reported adverse SRH outcomes, such as STIs, in the quantitative phase were asked about their experiences and subsequent actions in the qualitative phase. The lead author (AS) and a female RA conducted face-to-face interviews at private and convenient locations such as participants’ homes or weekly meeting venues. The RA received a two-day training based on a training manual that covered the objectives and interviewing techniques. Each training session lasted for 2 h. The interview guide was pre-tested with four PwDs to assess its clarity and comprehensibility. The interviews lasted from 45 to 70 min. Before each interview, the participants provided verbal and written consent. Qualitative data were collected between May 5 and July 11, 2022. Data saturation was achieved after interviewing the 35th participant. However, two more interviews were conducted with the interested individuals to ensure that no relevant information was missed. Therefore, the data collection ended after interviewing the 37th participant.

#### Qualitative data analysis

AS and RA transcribed all the audio recordings verbatim to ensure accuracy. The transcripts were de-identified to protect participant identities and imported into NVivo version 12 (QSR International, Ltd., Daresbury Cheshire, UK) for analysis. Braun and Clarke’s [56, 57] six-step reflexive thematic analysis approach was used. AS read the transcripts thoroughly under the supervision of KM-R and TIE to familiarise with the data. AS, KM-R, and TIE coded the data, collated the codes into key topics and generated themes. KM-R and TIE reviewed and examined the themes for interrelationships and relevance. The team defined, refined, and confirmed the generated themes. Illustrative quotes representing the themes were selected from the transcripts, along with demographic information, such as sex/gender, age, disability type, and district of the participants. The qualitative phase of the research adhered to the reporting guidelines outlined in the Consolidated Criteria for Reporting Qualitative Research (COREQ).

#### Trustworthiness

Trustworthiness was maintained following Lincoln and Guba’s key strategies [58], including credibility, dependability, confirmability, and transferability [59]. Credibility was ensured by providing well-trained interviewers (RAs) with necessary skills and knowledge. The interview guide was pre-tested, interviews were conducted at agreed-upon locations, and debriefing sessions were held regularly [59]. The local language Twi was used to facilitate participants’ expressions, and various strategies were employed to ensure honesty in responses, such as encouraging frankness and rephrasing questions [60]. Dependability was achieved through a detailed study protocol, meticulous data collection, record keeping, and coding accuracy verification by the research team [61]. Confirmability was attained through the supervision, and confirmation of themes by the entire research team. Triangulation techniques, including qualitative and quantitative data collection from different participants were used [59]. To enhance transferability, the study methodology has been comprehensively described, allowing for potential replication and follow-up by other researchers. The research design, study setting selection, and purposive selection technique were detailed to facilitate the study’s applicability to other similar contexts [59]. In addition to this, the reflexive thematic analysis process and its considerations [57] was employed to analyse the data. For example, the qualitative data was inductively coded to generate emergent themes.

#### Data integration

To mitigate the inherent weaknesses and biases associated with both the quantitative and qualitative approaches, a triangulation of the findings from both phases was employed. Quantitative studies involve statistical analysis to systematically examine data, aiming to minimise bias through objectivity [62]. While they may lack depth in interpreting research findings, they can yield empirical and statistically significant outcomes [63]. Qualitative studies, on the other hand, offer context-specific insights into human experiences, providing detailed understanding. Combining both methodologies allows for a comprehensive investigation, enhancing detail understanding of participants’ experiences and SRH outcomes. Inductive reflexive thematic analysis [56], guided by O’Cathain et al.’s [64] principles of data integration in mixed-methods studies, facilitated triangulation of findings from both study phases. This involved independent analysis, identification of phase-specific themes, linking relationships between phases for combined interpretation, and drawing synthesis of the findings as shown in Table 4.

## Results

### Quantitative results

#### Socio-demographic characteristics of respondents

Table 1 summarises the socio-demographic characteristics of the 402 respondents. Most respondents were male (51.5%), had visual impairment (57.7%), lived in urban areas (80%), belonged to the Akan ethnic group (82%), practiced Christianity (88.6%), and had subscribed to the NHIS (96.8%). Approximately one-third of the respondents were aged 60 years or older, had completed senior high school or tertiary education (34%) and were employed (50%).

**Table 1:** Multivariable analysis of factors associated with sexual behaviour among persons with disabilities

Variable	Demographic characteristics		Ever had sex	Early sexual initiation	Condom use at first sex	HIV testing
	**Frequency**	**Percentage**				
**Disability type**						
Physically challenged	170	42.3	Ref	Ref	Ref	Ref
Visually impaired	232	57.7	1.043[0.689,1.579]	0.959[0.518,1.775]	1.377[0.977,1.939]	1.05[0.619,1.782]
**Disability severity**						
Mild	160	39.80	Ref	Ref	Ref	Ref
Severe	242	60.20	0.63^*^[0.407,0.974]	1.084[0.619,1.900]	1.10[0.814,1.473]	1.52[0.935,2.469]
**Age (Years)**						
18–29	46	11.4	Ref	Ref	Ref	Ref
30–39	79	19.7	3.39^***^[1.893,6.060]	0.749[0.254,2.207]	1.153[0.637,2.087]	3.613^**^[1.462,8.929]
40–49	73	18.2	3.56^***^[1.985,6.392]	0.37[0.104,1.296]	1.08[0.570,2.056]	2.055[0.794,5.314]
50–59	89	22.1	6.74^***^[3.353,13.53]	0.468[0.145,1.505]	1.04[0.559,1.936]	1.41[0.54,3.69]
60 +	115	28.6	9.86^***^[4.330,22.45]	0.488[0.150,1.590]	1.366[0.712,2.618]	0.49[0.176,1.365]
**Sex**						
Female	195	48.5	Ref	Ref	Ref	Ref
Male	207	51.5	0.86[0.581,1.284]	0.480^*^[0.254,0.907]	1.20[0.860,1.675]	0.549^*^[0.329,0.916]
**Residence**						
Kumasi Metro	323	80.4	Ref	Ref	Ref	Ref
Offinso North	79	19.7	1.18[0.675,2.078]	0.65[0.280,1.506]	1.23[0.809,1.869]	1.71[0.900,3.256]
**Level of education**						
No formal education	68	16.9	Ref	Ref	Ref	Ref
Primary	66	16.4	1.14[0.604,2.166]	1.16[0.523,2.578]	1.161[0.651,2.071]	0.687[0.301,1.568]
JHS	130	32.3	1.23[0.703,2.113]	1.03[0.482,2.218]	0.70[0.426,1.151]	1.49[0.721,3.094]
SHS/Tertiary	138	34.3	1.64[0.924,2.910]	0.47[0.196,1.136]	0.48^**^[0.287,0.794]	2.42^*^ [1.160,5.029]
**Religious affiliation**						
Christian	356	88.6	Ref	Ref	Ref	Ref
Non-Christian	46	11.4	1.22[0.604,2.477]	1.079[0.363,3.210]	1.22[0.700,2.125]	0.383^*^[0.158,0.930]
**Ethnicity**						
Akan	330	82.1	Ref	Ref	Ref	Ref
Non-Akan	72	17.9	1.01[0.583,1.750]	0.525[0.192,1.435]	0.982[0.629,1.532]	0.65[0.326,1.307]
**Employment**						
Not working	199	49.5	Ref	Ref	Ref	Ref
Working	203	50.5	0.979[0.596,1.608]	0.94[0.453,1.937]	0.89[0.571,1.372]	1.14[0.600,2.160]
**Income (GHC)**						
0–99	203	50.50	Ref	Ref	Ref	Ref
100–299	102	25.37	1.42[0.832,2.416]	0.68[0.332,1.386]	1.20[0.766,1.888]	1.71[0.887,3.295]
300 +	97	24.13	1.250.697,2.238]	0.79[0.332,1.868]	1.25[0.786,1.979]	1.86[0.913,3.802]
**NHIS**						
No	13	3.2	Ref	Ref	Ref	Ref
Yes	389	96.8	0.999[0.401,2.488]	1.83[0.236,14.22]	0.99[0.429,2.277]	1.415[0.385,5.200]
**Household size**						
1–4	231	57.5	Ref	Ref	Ref	Ref
5 +	171	42.5	1.766^*^[1.117,2.791]	0.93[0.530,1.631]	1.07[0.785,1.457]	1.04[0.642,1.686]
**Duration to the nearest health facility**						
Less than 30 min	133	33.1	Ref	Ref	Ref	Ref
30–59 min	192	47.8	1.21[0.768,1.906]	1.11[0.570,2.153]	1.08[0.755,1.532]	1.52[0.864,2.668]
60 and above minutes	77	19.2	1.214[0.676,2.180]	1.064[0.479,2.364]	0.93[0.594,1.455]	2.362^*^[1.155,4.831]
**Marital status**			N/A			
Never married	97	24.1	–	Ref	Ref	Ref
Married	178	44.3	–	0.89[0.368,2.160]	0.91[0.592,1.390]	2.382^*^[1.222,4.641]
Separated/Widowed/Divorced	127	31.6	–	1.57[0.628,3.926]	0.99[0.608,1.601]	3.317^**^ [1.52,7.24]

Exponentiated coefficients; 95% confidence intervals in brackets

*Ref* Reference, *JHS* Junior High School, *SHS* Senior High School, *NHIS* National Health Insurance Scheme, *GHC* Ghana Cedis, *N/A* Not applicable

^*^p < 0.05

^**^p < 0.01

^***^p < 0.001

#### Sexual behaviour among persons with disabilities

**Fig. 1:**
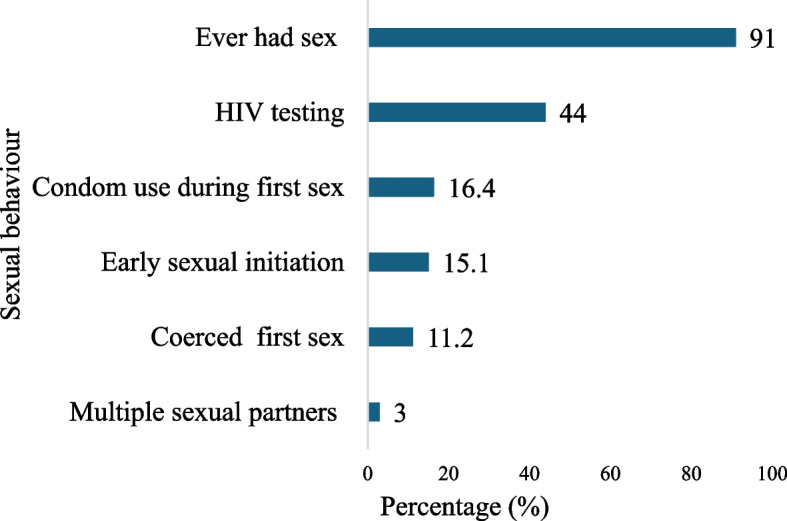
Sexual behaviour of persons with disabilities

Figure 1 shows the sexual behaviour of PwDs. Most (91%) of the respondents had ever had sex. About 16.4% did not use condoms during their first sexual encounter, and 3.0% had multiple sexual partners.

#### Factors associated with sexual behaviour among persons with disabilities

Table 1 presents the results of the multivariable analysis of the factors associated with sexual behaviours among PwDs. The severity of disability was negatively associated with the likelihood of having sex, as those with severe disabilities had lower odds of sexual intercourse than those with mild disabilities (aOR = 0.63; 95%CI = 0.407,0.974). Age was positively associated with the odds of engaging in sexual activity, as older respondents had higher odds of having sex than younger ones. Those who lived in large households (> 5 members) had higher odds of having sex than those who lived in smaller households (aOR = 1.766; 95%CI = 1.117,2.791). Males had lower odds of engaging in early sexual intercourse than females (aOR = 0.480; 95%CI = 0.254,0.907). Educational level was negatively associated with condom use during first sex, as those with higher educational levels had lower odds of using condoms than those with no formal education (aOR = 0.48; 95%CI = 0.287,0.794). HIV testing was positively associated with age, educational level, and travel time to the nearest health facility, as those who were older, more educated, and travelled longer had higher odds of testing than those who were younger, less educated, and travelled shorter. Marital status was also positively associated with HIV testing, as married individuals had higher odds of testing than those who had never married. However, HIV testing was negatively associated with gender and religion, as males and non-Christians had lower odds of testing than females and Christians respectively (Table 1).

#### Sexual and reproductive health outcomes

**Fig. 2:**
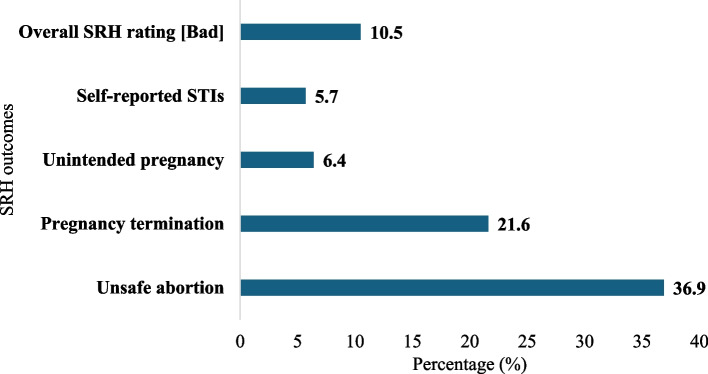
Sexual and Reproductive health outcomes among persons with disabilities

The results revealed that 10.5% of the respondents rated their SRH health status as bad while 5.7% reported having experienced an STI in their lifetime. The prevalence of unintended pregnancy was 6.4%. About 21.6% reported having terminated a previous pregnancy, and 36.9% of these terminations were unsafe (Fig. 2).

### Factors associated with sexual and reproductive health outcomes

Table 2 presents the results of the multivariable analysis of factors associated with SRH outcomes among PwDs. Educational level and NHIS subscription were negatively associated with unintended pregnancy, as those with higher education levels and NHIS subscription had lower odds of experiencing unintended pregnancy than those with no formal education and no NHIS subscription, respectively (aOR = 0.01; 95%CI = 0.11,0.82 and aOR = 0.02; 95%CI = 0.01,0.57). Self-rated SRH was positively associated with educational level, income level, and travel time to the nearest health facility, as those who had higher education levels, higher income levels, and longer travel time had higher odds of rating their SRH as good than those who had lower educational levels, lower income levels, and shorter travel time (aOR = 1.83; 95%CI = 1.08,3.09 for JHS; aOR = 1.74; 95%CI = 1.04,2.89 for SHS; aOR = 2.22; 95%CI = 1.19,4.12 for GHC300 + ; aOR = 2.02; 95% CI = 1.17,3.47 for > 60 min). Self-rated SRH was negatively associated with gender, as males had lower odds of rating their SRH as good than females (aOR = 0.55; 95%CI = 0.37,0.83).

**Table 2:** Multivariable analysis of sexual and reproductive health outcomes across demographic characteristics

Variable	Unintended Pregnancy	Pregnancy Termination	Sexually Transmitted Infections	Self-rated Sexual and Reproductive Health Status
**Disability type**				
Physically disabled	Ref	Ref	Ref	Ref
Visually impaired	4.00[0.27,56.59]	0.69[0.28,1.69]	0.96[0.36,2.59]	1.08[0.71,1.66]
**Disability severity**				
Mild	Ref	Ref	Ref	Ref
Severe	0.48[0.05,4.55]	0.64[0.29,1.41]	0.53[0.21,1.34]	0.79[0.55,1.14]
**Age (Years)**				
18–29	Ref	Ref	Ref	Ref
30–39	0.10[0.01,1.50]	1.16[0.21,6.47]	0.58[0.14,2.44]	1.40[0.62,3.18]
40–49	0.09[0.00,1.99]	2.28[0.39,13.21]	0.18[0.03,1.09]	0.81[0.38,1.74]
50–59	0.02^*^[0.10,0.62]	1.49[0.26,8.61]	0.32[0.06,1.81]	0.59[0.26,1.31]
60 +	N/A	1.93[0.30,12.29]	0.27[0.04,1.62]	0.56[0.25,1.26]
**Residence**				
Kumasi Metro	Ref	Ref	Ref	Ref
Offinso North	1.11[0.05,23.09]	0.74[0.25,2.15]	2.36[0.79,7.11]	0.66[0.42,1.01]
**Level of education**				
No formal education	Ref	Ref	Ref	Ref
Primary	0.06[0.01,2.41]	0.73[0.23,2.37]	1.28[0.16,10.20]	1.59[0.91,2.78]
JHS	0.07[0.00,1.01]	1.69[0.59,4.86]	1.78[0.34,9.40]	1.83^*^[1.08,3.09]
SHS/Tertiary	0.01^*^[0.11,0.82]	1.57[0.45,5.44]	1.72[0.34,8.80]	1.74^*^[1.04,2.89]
**Religious affiliation**				
Christian	Ref	Ref	Ref	Ref
Non-Christian	10.87[0.37,32.31]	1.87[0.39,8.93]	0.90[0.17,4.59]	1.69[0.88,3.24]
**Marital status**				
Never married	Ref	Ref	Ref	Ref
Married	1.52[0.05,43.09]	2.83[0.60,13.35]	2.85[0.79,10.26]	0.86[0.48,1.55]
Separated/Widowed/Divorced	0.97[0.03,29.33]	1.80[0.37,8.78]	0.82[0.11,5.80]	0.84[0.44,1.58]
**Ethnicity**				
Akan	Ref	Ref	Ref	Ref
Non-Akan	0.19[0.01,4.80]	0.89[0.27,2.87]	0.92[0.27,3.15]	0.55^*^[0.31,0.95]
**Employment**				
Not working	Ref	Ref	Ref	Ref
Working	08.81[1.20,29.86]	1.12[0.41,3.08]	0.81[0.23,2.85]	0.69[0.41,1.15]
**Income (GHC)**				
0–99	Ref	Ref	Ref	Ref
100–299	0.10[0.01,1.34]	1.46[0.54,3.94]	0.49[0.11,2.18]	2.34^**^[1.28,4.28]
300 +	0.02[0.01,1.02]	0.89[0.25,3.18]	1.61[0.43,6.07]	2.22^*^[1.19,4.12]
**NHIS**				
No	Ref	Ref	Ref	Ref
Yes	0.02^*^[0.01,0.57]	0.31[0.20,3.16]	0.51[0.10,2.56]	0.67[0.21,2.15]
**Household size**				
1–4	Ref	Ref	Ref	Ref
5 +	0.33[0.02,5.47]	0.89[0.40,2.03]	1.18[0.46,2.98]	0.97[0.67,1.40]
**Duration to the nearest health facility**				
Less than 30 min	Ref	Ref	Ref	Ref
30–59 min	0.47[0.03,8.19]	0.50[0.19,1.36]	0.67[0.23,1.95]	1.62^*^[1.10,2.39]
60 and above minutes	2.21[0.05,95.78]	1.14[0.37,3.48]	0.63[0.14,2.85]	2.02^*^[1.17,3.47]
**Sex**				
Female	N/A	N/A	Ref	Ref
Male	N/A	N/A	1.41[0.51,3.91]	0.55^**^[0.37,0.83]

Exponentiated coefficients; 95% confidence intervals in brackets

*Ref* Reference, *JHS* Junior High School, *SHS* Senior High School, *NHIS* National Health Insurance, *GHC* Ghana Cedis, *N/A* Not applicable

^*^p < 0.05

^**^p < 0.01

#### Interpersonal violence against persons with disabilities

**Fig. 3:**
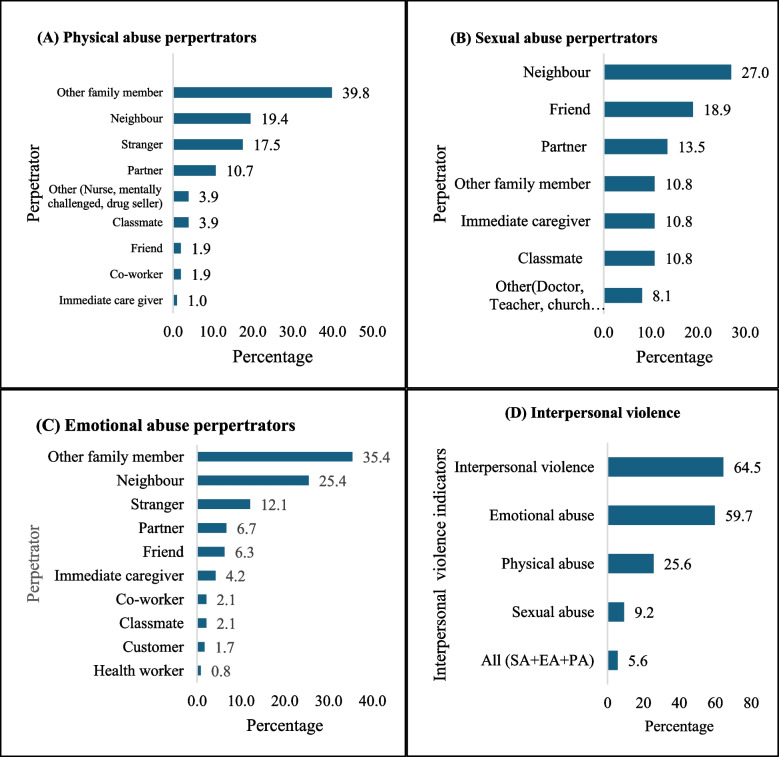
Prevalence of interpersonal violence and its perpetrators against persons with disabilities; SA = Sexual abuse; EA = Emotional abuse; PA = Physical abuse

Figure 3 displays the prevalence of different types of violence/abuse experienced by PwDs. The findings indicate that most PwDs (65%) have ever experienced at least one form of violence, while 6% have experienced sexual, emotional, and physical abuse simultaneously. The most common type of violence was emotional abuse (59.7%), followed by physical abuse (25.6%), and sexual abuse (9.2%). The main perpetrators of sexual violence were neighbours (27%), friends (18.9%), and partners (13.5%). For emotional abuse, the primary perpetrators were other family members (35.4%), neighbours (25.4%), and strangers (12.1%). For physical abuse, the main perpetrators were other family members (39.8%), neighbours (19.4%), strangers (17.5%), and partners (10.7%).

#### Factors associated with interpersonal violence against persons with disabilities

Table 3 presents the results of multivariable analysis of factors associated with IV against PwDs. The findings reveal that PwDs with visual impairment (aOR = 3.36; 95%CI = 1.90,5.93) and severe disability (aOR = 1.78; 95%CI = 1.09,2.92) had a higher likelihood of experiencing IV compared to those with physical disability and mild disability, respectively. Non-Christians (aOR = 0.39; 95%CI = 0.17,0.93) had lower odds of experiencing IV compared to Christians. Regarding income, PwDs earning GHC300 and above (aOR = 2.36; 95%CI = 1.09,5.09) had higher odds of experiencing IV compared to those earning less than GHC300. Additionally, PwDs living in large households (5 +) (aOR = 1.87; 95%CI = 1.13,3.10) had higher odds of experiencing IV compared to those living in small households (< 5).

**Table 3:** Multivariable analysis of factors associated with interpersonal violence against persons with disabilities

Variable	Emotional abuse	Sexual abuse	Physical abuse	Interpersonal violence
**Disability type**				
Physically challenged	Ref	Ref	Ref	Ref
Visually impaired	3.31^***^[1.89,5.80]	2.17[0.89,5.31]	0.70[0.40,1.22]	3.36^***^[1.90,5.93]
**Disability severity**				
Mild	Ref	Ref	Ref	Ref
Severe	2.29^***^[1.41,3.72]	1.01[0.45,2.24]	1.33[0.80,2.23]	1.78^*^[1.09,2.92]
**Age (Years)**				
18–29	Ref	Ref	Ref	Ref
30–39	1.94[0.80,4.67]	0.60[0.18,2.08]	1.14[0.46,2.83]	1.88[0.76,4.64]
40–49	2.80^*^[1.06,7.36]	1.04[0.29,3.74]	1.43[0.54,3.77]	2.39[0.88,6.48]
50–59	0.95[0.37,2.47]	0.28[0.06,1.37]	0.58[0.21,1.64]	0.67[0.25,1.78]
60 +	0.54[0.20,1.46]	0.58[0.12,2.73]	0.53[0.18,1.50]	0.37[0.13,1.01]
**Sex**				
Female	Ref	Ref	Ref	Ref
Male	1.42[0.84,2.39]	0.51[0.23,1.15]	1.39[0.81,2.38]	1.39[0.82,2.38]
**Residence**				
Kumasi Metro	Ref	Ref	Ref	Ref
Offinso North	1.31[0.67,2.55]	1.98[0.75,5.22]	1.14[0.58,2.21]	1.044[0.53,2.05]
**Level of education**				
No formal education	Ref	Ref	Ref	Ref
Primary	0.68[0.31,1.51]	1.52[0.29,7.82]	0.91[0.39,2.13]	0.84[0.38,1.89]
JHS	0.92[0.45,1.88]	2.09[0.51,8.55]	0.72[0.33,1.55]	1.09[0.53,2.23]
SHS/Tertiary	1.36[0.66,2.81]	2.57[0.65,10.10]	0.88[0.42,1.85]	1.56[0.75,3.25]
**Religious affiliation**				
Christian	Ref	Ref	Ref	Ref
Non-Christian	0.37^*^[0.16,0.87]	0.47[0.10,2.19]	1.04[0.45,2.41]	0.39^*^[0.17,0.93]
**Marital status**				
Never married	Ref	Ref	Ref	Ref
Married	1.52[0.78,2.98]	0.88[0.33,2.35]	1.54[0.77,3.08]	1.76[0.88,3.50]
Separated/Widowed/Divorced	1.62[0.75,3.50]	0.66[0.20,2.20]	1.65[0.72,3.75]	2.03[0.92,4.48]
**Ethnicity**				
Akan	Ref	Ref	Ref	Ref
Non-Akan	1.39[0.67,2.89]	1.29[0.47,3.56]	0.98[0.48,2.00]	1.23[0.58,2.62]
**Employment**				
Not working	Ref	Ref	Ref	Ref
Working	1.21[0.64,2.32]	2.41[0.80,7.24]	1.07[0.537,2.122]	1.372[0.717,2.625]
**Income (GHC)**				
0–99	Ref	Ref	Ref	Ref
100–299	2.97^**^[1.53,5.76]	0.74[0.22,2.49]	0.69[0.34,1.43]	2.38^*[^1.23,4.61]
300 +	2.61^*^[1.237,5.515]	1.91[0.66,5.50]	1.43[0.68,3.04]	2.36^*^[1.09,5.09]
**Number of people in household**				
1–4	Ref	Ref	Ref	Ref
5 or more	1.62[0.99,2.65]	0.67[0.31,1.53]	1.72^*^[1.029,2.858]	1.87^*^[1.13,3.10]
**Duration to the nearest health facility**				
Less than 30 min	Ref	Ref	Ref	Ref
30–59 min	1.41[0.79,2.50]	0.638 [0.27,1.52]	0.57[0.32,1.03]	1.137[0.63,2.04]
60 and above minutes	2.78^**^[1.34,5.77]	1.12[0.35,3.56]	0.92[0.45,1.91]	2.16^*^[1.03,4.56]

Exponentiated coefficients; 95% confidence intervals in brackets

*Ref* Reference, *NHIS* National Health Insurance Scheme, *GHC* Ghana Cedis

^*^p < 0.05

^**^p < 0.01

^***^p < 0.001

### Qualitative results

The qualitative phase of the study involved 37 participants (22 males and 15 females). Most of the participants resided in the Kumasi Metropolis (*n* = 24), belonged to the Akan ethnic group (*n* = 31), practiced Christianity (*n* = 34), and were married (*n* = 19). The remaining participants came from the Offinso North district (*n* = 13). The inductive thematic analysis revealed five main themes: a) curiosity to engage in sexual activities, b) feelings of despair and insecurity with abled partners, c) preference for sexual relationships with other PwDs, d) IV and its perpetrators, and e) adverse sexual and reproductive health outcomes. Each theme is discussed below with illustrative quotes from the participants.

### Theme one: Persons with disabilities curiosity to engage in sexual activities

The first theme that was derived from the participants’ sexual experiences was curiosity to engage in sexual activities. Some participants narrated their personal curiosity during their adolescence, a developmental phase characterised by diverse exploratory behaviours. This curiosity influenced some PwDs to engage in sex. Contrary to the common stereotype that PwDs are asexual [65], almost all participants reported having had sexual intercourse at some point in their lives which is a major component of sexual health [66].

“My first sex, I was curious. I wanted to know what it was like. I remember a friend told me that the thing is very nice. So, at age 16, I tried and the way the thing was painful, I couldn’t so I told him to stop. After that, any man that tries to convince me, I deny him. Because I thought to myself that I haven’t gotten to that stage yet and I didn’t want to get pregnant”. [Visually impaired, Kumasi, Female, 21 years]

“If I say that I have not had sexual intercourse before, then I would be a liar. But here in Africa, if you are a disabled person, people think that you should not have sex. And so, it is difficult to find a woman that you can have a relationship with”. [Physically disabled, Kumasi, Male, 36 years]

### Theme two: Feelings of despair and insecurity with abled partners

The second theme that was derived from the data was PwDs feelings of despair and insecurity with abled partners which affects age at first sexual encounter and number of sexual partners.

… I have no partner currently. Because they want sex not love. That is why I am not giving them the chance. You see right now, there is nothing that a man can tell me that another man has not told me before so whatever mindset you came with, I know this is what you want”. [Visually impaired, Kumasi, Female, 26 years]

#### Sub-theme: Age at first sexual intercourse

The participants also shared their experiences on the age of sexual debut and the reasons for initiating sex. The data revealed that the majority of the PwDs tend to start sexual activity later in life. Several participants reported having their first sexual intercourse at the age of 40 or older.

“So, I think that I started having sexual relationships after I was 40 years old. Our ancestors have a saying that, “If you start swimming early, your eyes will become red soon”. That means that if you start having sexual relationships early, you will have the consequence which might be an unintended pregnancy. Throughout my life, I have only had sexual relationships with two people. That is because I know myself and I know my background. So, I focused a lot on developing my skills and crafts so that I would be capable of caring for myself and the woman I am with. [Physically disabled, Offinso North, Male, 57 years]

Additionally, participants discussed how their religious beliefs influenced their sexual lives and their readiness to face the consequences of early sexual initiation. Besides, some participants emphasised the importance of obtaining consent and practicing safe sex during their first sexual encounters.

“I had my first sexual intercourse at age 31. I will say that it might be because of my upbringing. We are Muslims and my house is full of ‘Malams’. And so, if you get a girl, where will you take her? [laughs]”. [ Visually impaired, Kumasi, Male, 38 years]

“Yes, I have ever had sex. But it was a protected one with condoms. And each was aware. After we parted from each other I decided to stop those things because I wanted to be more serious for tertiary education so since that I have not had any serious relationship. My first affair with a lady was when I was 18 and I had only one sexual partner since I didn’t want to risk it for myself”. [ Visually impaired, Kumasi, Male, 25 years]

#### Sub-theme: Number of sexual partners

The participants also discussed the number of sexual partners they have had. While some participants described how their sexual relationships have changed over time. Other participants also reflected on their past sexual experiences, indicating how they had numerous sexual relations in the past. However, some of the participants also discussed how they are no more engaging in multiple concurrent sexual relations due to their responsibilities as parents. Others also discusses the financial strains associated with multiple sexual partners.

“…I have been involved sexually with two people before my current husband. So in all three people but not at the same time[laughs]”. [Visually impaired, Kumasi, Female, 45 years].

“…If I am not exaggerating, I can say that I have had at least ten sexual relationships in this place. However, I no longer engage in concurrent sexual relationships because I have two daughters that I must cater for. If I bring in another woman, then it is going to drain me financially”. [Visually impaired, Offinso North, Male, 49 years]

### Theme three: Preference for sexual relationships with other persons with disability

Some participants disclosed the challenges they encounter in finding sexual partners due to their disability status. Some participants therefore suggested preference for intra-disability relationships to increase their chances of finding love and understanding. Other participants also discussed the gratitude, understanding, and financial support from their partners with disability.

“So, my wife is the first woman I ever had sexual intercourse with. If you are disabled, it is difficult to get a woman to love you, unless you marry your fellow disabled person”. [Physically disabled, Offinso North, Male, 35 years]

“...Along the line, another man came for me. He was also blind. With him, I gave birth to just one…” [Visually impaired, Kumasi, Female, 60 years].

“I must admit that if it hadn’t been for this man that I had the child with [a visually impaired], then I would not have had any sexual intercourse in my life. I always had the notion that I have to get someone who would understand my situation and can also provide financially for my needs”. [Visually impaired, Offinso North, Female,33 years]

Despite this, a few of the participants also emphasised the importance they attach to physical support in their choice of sexual partners. Hence, they rather prefer to engage in relationship with people who are not disabled to assist them with their lives.

“…But if I am supposed to go for a woman to have a sexual relationship with, I will go for someone who is not disabled because I need someone to physically support me”. [Physically disabled, Kumasi, Male, 36 years]

“For personal reasons, I would prefer a person who has more sight than me so that the person can assist me. But not someone in my situation because I don’t know how the person can assist me”. [Visually impaired, Kumasi, Male, 25 years]

### Theme four: Interpersonal violence and its related perpetrators against persons with disability

The fourth major theme derived from the data was IV and its perpetrators against PwDs. Three sub-themes were identified under this theme: a) sexual abuse, b) emotional abuse, and d) physical abuse and the perpetrators of each type of violence against PwDs*.*

#### Sub-theme: Experiences related to sexual exploitation and abuse and its perpetrators against persons with disability

Most of the participants highlighted the widespread occurrence of sexual abuse against PwDs. While some participants did not disclose their personal experiences, they narrated incidents involving their fellow PwDs from both rural and urban settings. The fact that PwDs have substantial knowledge about sexual abuse among their peers suggest the high prevalence of such abuse against them. Moreover, many cases of sexual abuse remain unreported within communities due to socio-cultural and religious factors.

“I have heard about such sexual violence against women with disabilities. One of my colleagues called XXX claimed that she was sexually abused by a man who even impregnated her. The man is able while XXX is disabled. And so, when he was in the mood for sex, he raped her. The lady took the case to XX FM (Kumasi) for them to assist her. But I have never experienced anything of that sort”. [Physically disabled, Kumasi, Male, 36 years]

While some participants did not share their own experiences, they rather shared the experiences of other PwDs who have ever been abused.

“I have not been raped or sexually exploited. But I know a disabled girl who was sexually abused, and she got pregnant. There is another disabled girl, who is deaf who was also exploited, and she got pregnant. She has given birth to three children, but they were all because of sexual exploitation. It is difficult to know whether it is one person who is doing that to these girls in the community”. [Physically disabled, Offinso North, Female, 36 years]

#### Perpetrators of sexual abuse

Participants also described the various individuals who committed sexual abuse against PwDs. The data revealed that these perpetrators belonged to different segments of society, highlighting the vulnerability of PwDs to abuse across their entire communities. These perpetrators included intimate partners, family members, neighbours, and even professionals in positions of trust who abused their authority to exploit PwDs, especially females.

“I was at the age of 15 years, and I was sick, so I decided to go to the hospital for a check-up and there was this doctor who took advantage of me. When I got to the hospital facility and it was time for consultation, he told the person who assisted me to go out not knowing he was having some bad intentions. He started touching me here and there. I couldn’t defend myself, so he had sex with me. He put some fear in me that I shouldn’t tell anybody, and I was afraid so I couldn’t tell anybody about the incident. If not for this interview, I wouldn’t have said it to anyone or anywhere”. [Visually impaired, Kumasi, Female, 58 years]

Participants also discussed the gendered aspect of sexual abuse, indicating that in most cases, female survivors are more common. While some males considered attempts by females to sexually exploit them as beneficial, not all male PwDs viewed such actions as opportunities or ‘scholarship’.

"…For me, personally, no. But I know friends with disabilities who have been sexually exploited. It is not common for a woman to do that to a man. Mostly, it is the men who take advantage of the disability of the woman to have sex with her. When the women sexually coerce a man, it will normally lead to marriage. We say that the person has been given scholarship [laughs]” [Visually impaired, Kumasi, Male,38 years]

“…There are times too that I have visited female friends and they have touched me inappropriately. When I wasn’t completely blind, I went to a friend’s place, and she came from the bathroom almost naked. Thankfully, I was ‘morally’ afraid and so, I didn’t act on that temptation”. [Visually impaired, Kumasi, Male, 55 years]

#### Sub-theme: Experiences related to emotional abuse and its perpetrators against persons with disability

Another type of abuse disclosed by participants was emotional abuse. They described experiencing various forms of emotional abuses from different sources. Some recounted enduring emotional abuse within their intimate relationships, families, friendships, and professional interactions. Others mentioned relying on their faith in God to cope with their emotional challenges, as they felt no one else could provide relief from their current situation.

“Yes, I have experienced emotional abuse before. The father of my child has abused me emotionally because he has left the caregiving of the child to me. He does not support me in any way and that is emotionally draining. A lot of people have advised me to report him to social welfare, but I have not done so. I have left everything to the hands of God”. [Physically disabled, Kumasi, female, 33 years]

Another source of emotional abuse reported by participants was their spouses and family members. They described how their spouses prevented them from attending important social events including funerals due to the societal stigma attached to disability. Some participants also narrated instances where their family members emotionally mistreated them when they expressed their intentions to marry.

“Sometimes, there will be family gatherings or other social gatherings. But because of my condition, some people can look me in the face and tell me that I am not fit to attend those programmes. For example, my wife’s uncle died, and we had to go for the funeral, but she didn’t allow me to go because she thought I was not fit enough to show up at her uncle’s funeral. This is my wife’s uncle oh; so, you can imagine if her mother passes away. The family wanted to see me, but my wife felt ashamed and stopped me from attending. So, it is like you have been killed emotionally. It is not easy, oh! From that time, I got to know that she can neglect me at any time”. [Visually impaired, Kumasi, Male, 55 years]

“…That one, a lot of people do, including the family you are going to marry from and the people around you. They will be emotionally abusing you and be saying ‘PwD who do you think will marry you? Who do you think will love you? When you come to our family, you will bring a lot of disabled children’. So emotionally, we are always being abused. At a point when I decided to marry my wife, some family members were like you, you can’t see, how you can determine if this is a good lady or a bad lady. Not even my wife’s family, this is my own family. So, you see the kind of emotional abuse? They were like you can’t see so how can you plan your wedding? Do you think the lady can have sex with you? But I did everything about my wedding myself. Because when they speak like that, you begin to suspect them. So, these are some of the emotional abuse people go through but some of us [PwDs] because we are strong enough, we can overcome it but most people, especially those who are not educated, will not survive it”. [Visually impaired, Kumasi, Male, 31 years]

Another form of emotional abuse experienced by participants was the belittling and neglect from society and professionals. They described how they faced discrimination and ridicule from society, even when they demonstrated competence and confidence in their work. Furthermore, they reported that some people exacerbated this mistreatment by encouraging others to ignore or exclude PwDs, resulting in feelings of worthlessness or insignificance. Moreover, some PwDs reported that certain professionals who should exhibit professionalism sometimes subjected them to abuse which demotivated them to seek care.

“Some people really abuse us [PwDs] emotionally. Some people can tell me that I am like this because of my sins. A man brought his shoes for me to repair and after I did it, he refused to pay up. When I asked about it, he threatened to beat me and then insulted me that this is the reason why I am in this condition. This person eventually suffered a stroke for three years before dying”. [Physically disabled, Offinso North, Male, 46 years]

“A nurse at a hospital insulted me for getting pregnant simply because I am blind, and I had sex. So, sex should only be done by those who are not blind? What I did was that I reported her to those who were at the facility [other health workers], so they asked me whether I have seen the doctor, but I told them what happened, and I was very sad, so I just left the place without seeing the doctor”. [Visually impaired, Kumasi, Female, 58 years]

#### Sub-theme: Experiences related to physical abuse and its perpetrators against persons with disability

This sub-theme explored the physical abuse and its perpetrators against PwDs. They recounted experiences of suffering physical abuse from intimate partners, extended family members, strangers, and general community members. Additionally, participants discussed the consequences of the abuse they endured, particularly during childbirth. Some participants chose to avoid physical violence by ending relationships through divorce, while others relocated to escape abuse from family members. Despite the empowerment displayed by some participants, a few believed that being a PwD required passivity, perceiving themselves as physically weaker than those without disabilities. Consequently, they felt compelled to remain humble and avoid confrontations to prevent harm.

“…I mean the fourth born when I gave birth to him, the hand wasn’t functional. It was this very man [partner] who pushed me to the ground when I was pregnant. So, when I gave birth to him, this hand wasn’t functional”. [Visually impaired, Kumasi, Female, 60 years]

“My husband has abused me physically before... He is not the only person who did that to me. My first son’s father also abused me physically and that’s what brought about our divorce”. [Physically disabled, Kumasi, Female, 39 years]

“I think that because you are disabled, you have to be submissive because you do not have the strength of persons without disabilities. Once you plead and try to avoid confrontations, then you can prevent any physical violence”. [Physically disabled, Kumasi, Male,27 years]

### Theme five: Adverse sexual and reproductive health outcomes experienced by persons with disabilities

The fifth theme that was generated from the data was the adverse sexual and reproductive health outcomes experienced by PwDs. Three sub-themes were identified under this theme: a) unintended pregnancy and navigating delicate decisions about pregnancy termination, b) self-reported sexually transmitted infections and navigating challenges to seek care, and c) perception on personal sexual and reproductive health.

#### Sub-theme: Unintended pregnancy and navigating delicate decisions about pregnancy termination

This sub-theme explored the experiences of PwDs regarding unintended pregnancy. Some of the participants reported that they had unintended pregnancy that were compounded by their disability status. Nonetheless, they decided to carry their pregnancies to term. Other participants had to face difficult decisions about whether to terminate their unintended pregnancies or not, considering the social and moral implications of their choices.

“…I have ever had unintended pregnancy before. I was actually in a sexual relationship with the man though. He had proposed to me, and I had accepted to be in a relationship with him. But it looks like he was not ready to have a child because he was already married with another woman. So, he didn’t want his wife to get to know about my pregnancy”. [Physically disabled, Offinso North, Female, 36 years]

“For the first man I had a child with, he initially denied responsibility for the child because someone had told him that because I am disabled, if he accepts the pregnancy, then I will die during delivery. But after I gave birth, he now came to claim responsibility over the child”. [Physically disabled, Offinso North, Female, 60 years]

“Yes, I have terminated pregnancy once in my lifetime. At that time, I was a teacher by then. So, this man came into my life, and I later got to know that he was having a wife and my family is a respectful one. People around me also hold me in high esteem because they know how I have carried myself. So, I needed to terminate it even though I didn’t like the idea”. [Visually impaired, Kumasi, Female, 45 years]

#### Sub-theme: Self-reported sexually transmitted infections and navigating challenges to seek care

This sub-theme examined the experiences of PwDs regarding STIs and how they sought care for them. Both male and female participants reported having personal experiences with STIs and accessing various formal and informal sources of SRH care to treat their infections. Other participants also reflected on the causes of their STIs and the need to adopt preventive measures to avoid similar situations in the future. These experiences underscore the importance of personal choices, consequences, and the pursuit of healthier practices.

“I have ever had an STI before. I once cheated on my wife and ended up with gonorrhoea. I woke up with severe pains whenever I was passing urine. So, I went to my friend who was a pharmacist, and she gave me some medications. Since then, I have been faithful and not experienced any STIs”. [Physically disable, Kumasi, Male, 47 years]

“I had chlamydia some time ago. And so, I went to the hospital for them to help me treat it. At the facility, I was treated and then they educated me on douching because it was something that could put you at higher risk of chlamydia”. [Visually impaired, Offinso North, Female, 51 years]

“I had gonorrhoea some time ago. Fortunately for me, I was able to detect it early. I was going to the farm with my old man, and I told him that lately, I feel a lot of pain when I urinate. So, he asked me whether I have had sex with anyone lately...”. [Visually impaired, Offinso North, Male, 49 years

#### Sub-theme: Perception on sexual and reproductive health status

This sub-theme explored the self-rated SRH of PwDs. Two main contrasting views were expressed regarding the SRH of PwDs. While some participants rated their SRH as good, others rated theirs as bad. Some participants expressed contentment and satisfaction with their reproductive health, emphasising trouble-free childbirth experiences. This positivity contributed to their positive perception of their reproductive health. Positive perception was evident in their high self-ratings, marked as “very good,” associated with well-being and absence of STIs. Generally, individual perspectives varied, others emphasising successful childbirth, while others focused on STI-free well-being.

“I will rate it as very good because I have no problem relating to my reproductive health and system. I have been able to give birth without any complications”. [Physically disabled, Kumasi, Female, 49 years]

“I think that my reproductive health is good because I have not experienced any sexually transmitted infection. So, I will rate my sexual and reproductive health status as very good. I am very healthy. The last time I went to the hospital was barely a year ago and that was because I was feeling body pains”. [Visually impaired, Offinso North, Female, 54 years]

However, some participants, both male and female, indicated dissatisfaction with their SRH. Some males reported problems with erection, while females expressed frustration due to inability to engage in sexual activity, desiring medical interventions. Additionally, some highlighted painful menstruation, shortening their cycle from seven to three days, affecting their negative perception of their SRH.

“It is not good my sister! I remember that about six years ago, I went to the Volta region where I met a lady that I proposed to. When we went inside the room to have sex, I could not have an erection. I tried everything but I could not have an erection. The lady did everything to arouse me but my penis could not harden. I was really embarrassed that day. So, I bought some herbal medicine which changed things for me. I was now able to have an erection. So, for me, I think that my SRH is good. I can last longer in bed now”. [Physically disabled, Kumasi, Male, 36 years]

“I will say that it is not so good because as I said, I cannot have sex because of my condition. I wish there was a medication or treatment that could help me to have sex. I experience excruciating pains whenever I menstruate. Because of that, I don’t get seven days of menses but rather three days”. [Physically disabled, Kumasi, Female, 33 years]

### Triangulation

Table 4 summarises the integration and synthesis of qualitative interview findings and quantitative survey results.

**Table 4:** Triangulation of study findings

Themes	Sub-issue	Quantitative findings	Illustrative quote	Synthesis
Theme 1: Curiosity to engage in sexual activities Theme 2: feelings of despair and insecurity with abled partners which affects age at first sexual encounter and number of sexual partners Theme three: Preference for sexual relationships with other persons with disability	Persons with disabilities engagement in sexual intercourse	–91% indicated that they have ever had sex –9% never had sex	*“I must admit that if it hadn’t been for this man that I had the child with, then I would not have had any sexual intercourse in my life. I always thought that I must get someone who would understand my situation and provide financially for my needs. We had sex only twice. At that time, he was getting separated from his wife. So, our affair happened in his house because his son was the one picking me up for our group meetings. So, the mother kept enquiring from the son if he was aware of our affair”. [Visually impaired, Offinso North, Female, 33 years]*	The findings show that most PwDs have ever had sex. Nonetheless, some personal factors, such as age, religion, curiosity, and consent, influence their decision to engage in sexual practices. This highlights the need for comprehensive sexual education for PwDs to enable them to make informed decisions about their sexuality and sexual health.
	Age at first sexual intercourse	–15.1% had sex before 18 –84.9% had sex at or after 18 years	*“I was about 17 or 18 years old when I first had sex. I have been married for over 20 years”. [Physically disabled, Kumasi, Male, 43 years]* *“Frankly speaking, I think it was at the age of 25 upwards that I had my first sex. You know; when we are in school, they tell us that it is not good to have sex. But sometimes, you will meet someone who wants to help you in some ways. I cannot remember the duration of the first sexual relation I had because it was a very long time ago. But that relationship ended before I came into a relationship with the father of my children”. [Visually impaired, Offinso North, Female, 51 years].*	The findings reveal the experiences and perceptions of sexual activity across different life stages among PwDs. The narrative demonstrates the complex interplay between societal norms, individual desires, and relationships. Various factors, such as age, religion, curiosity, and consent, influence sexual initiation suggesting that sexual decisions are often shaped by contextual dynamics. This also emphasises the need for comprehensive sexual education that provides accurate information about relationships, consent, and responsible decision-making.
	Number of sexual partners	**–**3% had multiple sexual partners **–**97% had 1 sexual partner	*“… That means that I have had sex with four women in my lifetime”. [Physically disabled, Kumai, Male, 36 years]* *“I have had it with only one person. I stayed with the man for almost 3 years before we broke up”. [Physically disabled, Kumasi, Female, 27 years]*	The results showed that most PwDs do not have multiple sexual partners but have changed sexual partners over time. This reveals the challenges some PwDs experience in sexual relations which could also expose them to various STIs if they do not practice safe sex.
Theme 4: Interpersonal violence and its perpetrators against persons with disability	Sexual exploitation and abuse	**–**9.2% experienced sexual violence **–**Physically challenged = 7.65% **–**Visually impaired = 10.34% **–**Females = 11.28% **–**Males = 7.25%	*“There is this girl who is staying with her uncle. She is blind and this same uncle sexually abuses the blind girl, so it became a family issue but what the family said was mind blowing. They said that if the girl reports the case, it will tarnish the image of the family and her uncle was the one taking care of her too so she can’t tell anyone. Meanwhile, sexually, and emotionally, she has been abused”. [Visually impaired, Kumasi, Female, 45 years]* *“Yes, I have experienced sexual violence before. There was this girl who was also cohabiting with a man who was now recovering from injury. She found out the man was standing outside with another woman. And so, she had planned to take revenge for that. They visited me together on one occasion and later the woman came alone. I asked her why she came here alone, and she told me that she was in the neighbourhood and so decided to pass by. Later, she was insisting that she wanted to have sex with me to pay back the man. But I refused and she stopped”. [Physically disabled, Offinso North, Male, 48 years]*	The results emphasise the need for improved awareness, education, and support networks to protect the rights and well-being of PwDs. Addressing this issue requires a comprehensive approach, involving sensitizing communities and institutions about the rights and needs of PwDs. It also requires strengthening mechanisms to report and address cases of abuse and violence, ensuring that PwDs can access justice and support without fear of repercussions. Ultimately, creating an inclusive and supportive environment is crucial for safeguarding the sexual and reproductive health of PwDs.
	Emotional abuse	–59.7% experienced EV –Physically challenged = 50% –Visually impaired = 66.81 –Males = 64.73% –Female = 54.36%	*“Sometimes, they go to the extent of insulting me because of my situation[disability]. As a result, even the children in our house do not show me respect. But what can I do? Even Jesus Christ suffered such abuse from those close to me; how much more me? I have comforted myself. All I ask for is for my relatives to treat me fairly. They should just give what is due to me from our cocoa plantation. [Visually impaired, Offinso North, Male, 58 years]* *“Oh, yes! I have experienced emotional abuse even from my own biological sister. She did something and I talked about it. Because I reproached her, she started insulting me. She said to my face that I have crooked legs and that is why I am disabled. I got infuriated and cursed her because I was not born like this. Now, when she gave birth, her son was physically disabled. Since then, my sister could not abuse me because of my condition again”. [Physically disabled, Offinso North, Female, 46 years]*	The findings underscore the need to promote understanding, empathy, and respectful behaviour toward PwDs. This can be achieved by raising awareness, providing education, and creating support networks for PwDs and their communities. It can also be facilitated by challenging the stereotypes, stigma, and discrimination that PwDs face in various aspects of their lives. By fostering a culture of inclusion and respect, the rights and well-being of PwDs can be protected and enhanced.
	Physical abuse	–25.6% experienced PV –Physically challenged = 28.82% –Visually impaired = 23.28% –Males = 29.47% –Females = 21.54%	*“My husband has abused me physically before. We had a little argument and he hit me with his slippers. I decided to go to the hospital for treatment and after that go straight to the police station for a report, but I stopped because I saw that it wasn’t necessary. He is not the only person who did that to me. My first son’s father also abused me physically and that’s what brought about our divorce”. [Physically disabled, Kumasi, Female, 39 years]* *“… people sometimes think I’m inferior because of my disability or that they can physically beat me. About two months ago, I took a car to the stadium for training. The driver cheated me and refused to give me the correct change. When we reached Asafo, the driver and conductor tried to force me out of the car. The conductor even tried to hit me with a stone. In self-defence, I punched the driver and pushed the conductor to the ground. I threatened them with my walking stick, and they reported the case to the Asokwa police station. The police took our statements, and I had to pay for the damaged windscreen. My brother, coach, and elder helped with the payment. They also demanded bail money of GHC100 and treated me poorly, denying me the opportunity to eat that day”. [Physically Disabled, Kumasi, Male, 36 years]*	The results reveal the vulnerability of PwDs to various forms of abuse and mistreatment, including physical violence. PwDs already face numerous barriers in accessing appropriate healthcare, information, and support for their SRH. When abuse and mistreatment are added to these barriers, their overall health and well-being are further jeopardised. Ensuring the safety and well-being of PwDs should be a priority. This involves providing accessible resources and support systems for those who experience abuse. Additionally, law enforcement agencies and societal institutions should be equipped to handle cases involving PwDs sensitively and justly. Ultimately, the results indicate a critical need for a more inclusive and supportive society that upholds the rights and dignity of all individuals, regardless of their abilities.
Theme 5: Adverse sexual and reproductive health outcomes among persons with disability	Unintended pregnancy	**–**6.4% experienced unintended pregnancy	*“No! We did not plan the pregnancy; it was unintended. I was waiting for my menses to come but I missed it. That was what prompted me to check what was wrong with me”. [Visually impaired, Offinso North, Female,33 years]*	The results show that PwDs have an unmet need for some SRH services, such as contraception and family planning. Therefore, comprehensive SRH education can empower them to make informed decisions about their fertility and family size, contributing to their overall well-being and autonomy in managing their SRH.
	Pregnancy termination	**–**21.6% of unintended pregnancies were terminated	*“Yes, I have terminated pregnancy once in my lifetime. At that time, I was a teacher by then. So, this man came into my life, and I later got to know that he was having a wife and my family is a respectful one. People around me also hold me in high esteem because they know how I have carried myself. So, I needed to terminate it even though I didn’t like the idea”. [Visually impaired, Kumasi, female, 45 years]*	The findings have revealed that PwDs should have the same access to reproductive healthcare services as anyone else. Additionally, it stresses the importance of supporting PwDs in making choices that align with their personal values and circumstances, especially when they face complex situations like unintended pregnancies.
	Self-reported STIs	**–**5.7% self-reported STIs **–**Females = 4.10% **–**Males = 7.25%	*“When you come to the school, we are using the flushing toilets so at times you experience candidiasis. So when I go home, I try the herbal medicines like the Prekese [Tetrapleura tetraptera], garlic and those stuff and it goes”. [Visually impaired, Kumasi, Female, 21 years]* *“When it comes to sexually transmitted infections, I have ever had one before. I think I got infected because of the infidel act of my partner with a fellow church member. A friend of mine recommended some herbal medicine to me. I was feeling pain in my penis. I also had rashes around my penis, so when I told my friend, he told me that the man had gonorrhoea”. [Physically disabled, Offinso North, Male, 35 years]*	The data highlights the need for sexual health education and resources that are tailored to the needs of PwDs. It is essential that PwDs are informed about safe sexual practices, the importance of early detection and appropriate treatment of STIs and have access to SRH services without barriers. This can improve their SRH outcomes and well-being, as well as prevent further complications and infections.
	Self-rated SRH	89.5% good 10.5% bad	*“It is not so good because when I had my accident, I had a spinal injury that has affected every aspect of my life including my sex life. Moreover, I am growing old and so, I don’t have much strength like I used to some years ago”. [Physically disabled, Kumasi, Male, 35 years]* *“I will say my sexual and reproductive health status is not good because all is not well at all with me. Sometimes I experience pains in my reproductive system and abdomen”. [Visually impaired, Kumasi, Female, 45 years]*	Improving the SRH of PwDs requires a holistic approach that encompasses accessible healthcare, comprehensive education, and supportive services that address the unique challenges they may encounter. This approach should consider the diverse needs and preferences of PwDs, as well as the barriers and opportunities they face in accessing SRH information and care. This approach should also involve empowering PwDs to make informed and autonomous decisions about their SRH, as well as protecting their rights and dignity from abuse and discrimination.

## Discussion

### Summary of key findings

This study presents the results of a mixed-methods study that explored the sexual lives, experiences and reproductive health outcomes among PwDs in two districts of Ghana. The quantitative results showed that most PwDs have ever had sex. Sexual activity was associated with disability severity and age. About 16% did not use condom during their first sex and this was influenced by educational level, with those with secondary or higher level having the lowest odds. The results also showed that some PwDs had sex before age 18 years, with males being less likely to engage in early sexual intercourse compared to females. The results further showed that 3% had multiple sexual partners. It was also revealed that 44% had ever tested for their HIV status, with those aged 30–39, higher education, and married individuals having higher odds. However, males have lower odds of testing compared to females.

The majority (89.5%) rated their SRH status as good. Individuals with junior high school and senior high school educational levels, those earning GHC300 and above, and those with a duration to the nearest health facility over 60 min have higher odds to rate their SRH as good. However, males and non-Akans were less likely to self-rate their SRH as good. The results also showed that 5.7% indicated they had ever had an STI. It was also found that 6.4% of women with disability experienced unintended pregnancies and this was associated with SHS/tertiary education levels and NHIS subscription.

Approximately 21.6% had ever terminated a pregnancy, with 36.9% of these terminations being unsafe. About 65% of PwDs had experienced IV. Specifically, 59.7%, 25.6%, and 9.2% had experienced emotional, physical, and sexual violence, respectively. The visually impaired and those with severe disabilities, individuals earning GHC300 and above, those living in large households have higher odds of being abused. The qualitative data revealed five themes. These comprised curiosity to engage in sexual activities, feelings of despair and insecurity with abled partners, preference for sexual relationships with other PwDs, IV and its perpetrators, and adverse sexual and reproductive health outcome. The findings have implications for policy and practice regarding the SRH needs and rights of PwDs in Ghana. They suggest the need for accessible and comprehensive SRH education and services for PwDs, as well as mechanisms to prevent and address cases of IV against them. They also highlight the importance of empowering PwDs to make informed and autonomous decisions about their SRH, as well as fostering a supportive and inclusive environment for them.

### Synthesis with previous evidence

#### Sexual lives and experiences of persons with disability

The majority (91%) of the respondents indicated that they have ever engaged in sexual activity, contradicting the general notion that PwDs are asexual [65]. Previous studies in several LMICs including Burkina Faso [67], Cameroon (80%) [68], Haiti (49%), Rwanda (53%), Timor-Leste (51%), and Uganda (53%) [69] and Ethiopia (59.9%) [39] have reported high prevalence of sexual activity among PwDs. In this study, individuals with severe disabilities showed a reduced likelihood of engaging in sexual intercourse compared to those with mild disabilities, while age was positively associated with the odds of ever engaging in sexual activity, consistent with findings from previous studies [30, 34, 70]. Other studies found high rates of sexual activity among women with disabilities, young people with disabilities in Ethiopia [71, 72], adult women in Sierra Leone [73], and visually impaired and deaf adult women in Ethiopia [74]. However, a low rate of sexual activity (48%) was found among young people in Uganda [35]. Nevertheless, a scoping review in LMICs highlights the neglect of disability and sexuality, indicating the need for further exploration in this domain [12]. Possible reasons for the reduced likelihood of engaging in sexual activity among individuals with severe disabilities could be cultural beliefs, stigma, self-stigma, and discrimination attached to disability.

The study found that 15.1% had their first sexual intercourse before age 18, and males were less likely to engage in early sexual intercourse compared to females. The qualitative findings revealed that the majority of PwDs initiated their sexual life around age 30 to 40 years due to factors such as difficulty finding sexual partners and the perception that having a sexual partner would increase their burden. This highlights the necessity of sexual education for PwDs and self-efficacy. Additionally, the study found that 3.0% had multiple sexual partners, which was similar to what was reported as 1.4% in Uganda [75]. But the prevalence is lower than the 12.5% reported in Burkina Faso [67, 76] and 58.6% reported in Ethiopia [71]. Qualitative findings indicated that although some participants had more sexual partners in their lifetime, most of these relationships were not concurrent. Such relationships expose PwDs to different sexual partners and increase their risk of contracting STIs, including HIV and AIDS [67, 76].

Approximately 16.4% did not use condoms during their first sexual experience. Safe initiation of sexual behaviours is usually recommended and the possibility of continuing with these behaviours are high. However, individuals with SHS or higher educational levels had lower odds of using condoms during their first sexual encounter than those with no formal education. Previous studies have also reported low condom usage among PwDs, ranging from 22 to 37% [67, 70]. Yet, this study’s finding is slightly lower than what was reported in previous studies [67, 70]. Inconsistent results may be attributed to factors such as the time considered, study population, and gender disparities in contraceptive options. This finding highlights the need for increased education and behavioural change communication to promote safe sexual practices among PwDs. The findings have implications for policy and practice regarding the SRH needs and rights of PwDs in Ghana. They suggest the need for accessible and comprehensive SRH education and services for PwDs, as well as mechanisms to prevent and address cases of IV against them. They also highlight the importance of empowering PwDs to make informed and autonomous decisions about their SRH, as well as fostering a supportive and inclusive environment for them.

The study revealed that only 44% of the respondents had ever tested for their HIV status. The odds of testing were higher for PwDs aged 30–39, senior high school or tertiary and married individuals, but lower for males than females. These findings are consistent with previous studies that reported similar prevalence rates of HIV testing among PwDs in LMICs and suggested that demographic characteristics influence sexual behaviours of PwDs, including HIV testing [72, 77, 78]. The health outcome model [40–42] provides a theoretical explanation for the associations between age, marital status, and Christianity with HIV testing, as it posits that individual characteristics affect health outcomes. According to this model, as PwDs age, they are more likely to marry, and some marriages, especially Christian marriages, require HIV testing. Moreover, the model suggests that higher educational level enhances health awareness and behaviours, such as knowing one’s HIV status [79].

#### Interpersonal violence and its perpetrators against persons with disabilities

The findings indicate that 65% of PwDs had experienced some form of violence, with 6% facing combined sexual, emotional, and physical abuses. Emotional abuse was the most prevalent (59.7%), followed by physical abuse (25.6%) and sexual abuse (9.2%). While males reported more emotional violence, females experienced more sexual violence. These findings are consistent with Opoku et al.’s [21] rate of 68.3% in Ghana but higher than Hughes et al.’s [80] rate of 24.3%. Individual characteristics, as suggested by the health outcomes model, influenced violence experiences, especially for those with visual impairments and severe disabilities. This is in line with studies in several LMICs [81–86] that reported similar associations. The study addresses a critical aspect of the 2030 Agenda by emphasising disability-disaggregated data to ensure inclusivity. However, most existing research on violence against PwDs focuses on women and sexual violence [87–89]. Therefore, these findings underscore the need for inclusive violence prevention and response programs since IV is mostly a continuum [53], regardless of gender.

The study also showed that household size, disability type, and income level were associated with violence experiences. Larger households appear to expose PwDs to higher violence risk than smaller households. Both qualitative and quantitative findings indicate that family members, neighbours, and strangers, including intimate partners, are major perpetrators of emotional and physical violence [19, 87, 90]. Despite this, most survivors do not report incidents, echoing prior studies that this is especially true for survivors of sexual violence, particularly women [19, 87, 90].

Those with visual impairments and severe disabilities were more likely to experience violence than those with physical impairments. Previous studies have reported similar findings [19, 81]. The possible explanation is that these categories of PwDs rely on others for most of their daily activities compared to the physically impaired. The study further unveils that higher-income PwDs are more susceptible to violence, consistent with research indicating that PwDs who earn more than their partners may experience violence [20]. This could be due to partners or family members seeking control over economic resources, potentially leading to physical and emotional violence. The qualitative phase highlighted the widespread nature of violence across societal levels. Nonetheless, individuals in trusted positions like doctors, police, and teachers may misuse their authority to perpetrate violence, especially against women with disabilities. This underscores PwDs’ vulnerability to violence and emphasises the necessity for multifaceted efforts to address this issue.

#### Sexual and reproductive health outcomes among persons with disability

The study also found a 6.4% prevalence of unintended pregnancy among women with disabilities. This rate varies across different LMICs, as shown by comparable studies that reported prevalence rates ranging from 9.2% in Haiti to 47.1% in Mali, 28.8% in Nigeria, 29.8% in Pakistan, 23.0% in Rwanda, 26.9% in Senegal, 12.0% in South Africa, and 24.7%-40% in Uganda [35] and from 15.4% to 67% in Ethiopia [22, 74, 91]. A higher rate of 53% was reported in the USA [32]. The qualitative findings corroborated the quantitative results and indicated that unintended pregnancy was a common occurrence among PwDs. The multivariable analysis indicated that individuals with SHS or tertiary education and NHIS subscription had lower odds of experiencing unintended pregnancy. This supports the health outcomes framework which states that individual characteristics and health policies influence health outcomes [40–42]. For example, those who are educated are exposed to various strategies to prevent unintended pregnancy including abstinence and contraceptive usage. With NHIS subscribers, they can easily seek SRH services which could prevent them from unintended pregnancy. Some unintended pregnancies resulted in pregnancy termination, with 21.6% of respondents reporting termination experiences. However, 36.9% of these procedures were self-reported to be conducted unsafely, highlighting the need for education and interventions to promote appropriate medical care for pregnancy-related care. This implies that unintended pregnancy is a major issue among PwDs globally calling for measures to improve appropriate contraceptive use among PwDs.

The prevalence of self-reported STIs in this study was 5.7%. Participants self-reported STIs such as gonorrhoea, syphilis, HIV and Chlamydia, in the qualitative data. This prevalence is comparable to the rates reported among the general male population in Ghana (6.0%) [92] and 3.8% in sub-Saharan Africa [93]. Previous studies among PwDs in LMICs found varying STIs-HIV prevalence rates ranging from 0.6% in Niger to 25.3% in Ethiopia [68, 76, 94]. A systematic review on HIV among PwDs also found prevalence rates between 1.1% and 29% [95]. The differences in the study findings could be due to variations in the study population, time, and measurement of the outcome variables. These findings imply the urgent need to demystify the general assumption that PwDs do not have sex [65] which negatively impacts their sexual behaviour and use of SRH services increasing their risk of STIs, including HIV and AIDS [37]. Nonetheless, the prevalence of self-reported STIs in this study could be due to underreporting and social desirability biases. For example, only 44% indicated that they have ever tested for their HIV status. Research indicates that PwDs often hold misconceptions about their risk of STIs exposure and may avoid accessing STI services due to the fear of stigma [75, 96]. These challenges are exacerbated by the lack of disability awareness and training among healthcare providers, limited access to STIs testing information and services, and communication barriers [78, 97].

About 10.5% of PwDs rated their SRH as bad/poor. Self-rated SRH was associated with education, income, duration to the health facility and sex. This finding is consistent with the prevalence reported in previous Ghanaian studies [98, 99]. Discussing the findings within the health outcomes model shows that those with high level of education and income had higher odds of reporting good SRH status [40–42]. Those who are educated are more likely to recognise health problems and seek prompt care when needed. In addition, recognising the need to access healthcare alone is not enough, but money is required to pay the cost associated with health seeking. Distance to the health facility has various impacts on the health and health seeking behaviour of PwDs. The literature indicates rich people can afford healthcare more easily, leading to better healthcare access and longer life expectancy [99, 100]. High income enables the ability to purchase healthcare services, and research has shown that it also provides a favourable living environment that positively impacts health [99, 101]. The findings on the association between sex and self-rated SRH is different from what was reported by Debpuur et al. [98]. They found that women were more likely to self-rate their health status as bad. The possible explanation for the differences in study findings could be that females are more likely to notice SRH problems due to their reproductive make-up and seek more SRH services compared to men [102].

### Implications for policy and practice

The study findings have significant practical and policy implications. First, the results reveal that most PwDs have ever had sex, but misconceptions about their sexuality and fear of stigma hinder their access to SRH services, including HIV testing [75, 96]. Addressing these issues requires behavioural change programs to promote safe sexual practices and encourage PwDs to seek SRH for STIs and safe abortion services.

Secondly, the findings indicate that both male and female PwDs experience various forms of violence. Interventions to combat violence against PwDs should target both genders and raise awareness of the negative effects of violence. Additionally, self-stigma and societal normalisation of violence against PwDs should be addressed through intensified education for PwDs and community members. The perpetrators of violence against PwDs come from various sources within the community. This highlights the vulnerability of PwDs to violence at all levels of society, necessitating immediate and pragmatic interventions to protect them. Therefore, national policies, such as the Disability Act 715, the criminal justice system, and the health system, should adopt systematic and standardised methods for assessing and recording possible violence against PwDs. Additionally, the reluctance of PwDs to disclose violence due to a lack of awareness of their rights, fear of losing social support, and disability-related financial assistance should be acknowledged and addressed.

Thirdly, the prevalence of adverse SRH outcomes, such as STIs, unplanned pregnancy, and unsafe abortion, underscores the need for targeted efforts and the development of new interventions to address these issues effectively. Finally, the SRH of PwDs is a growing public health concern, while a majority of PwDs rated their SRH status as good, many still reported experiencing various SRH problems, including STIs and sexual dysfunction. This study indicates that individuals with higher wealth and higher levels of education experience better SRH compared to those with lower economic status. Hence, it is essential to prioritise the SRH, education and economic security of PwDs. Government is encouraged to enhance economic and social integration through policy decisions. For instance, providing more formal education and economic support to PwDs can improve their socio-economic status and positively impact their SRH.

### Strength and limitations

The combined analysis of quantitative and qualitative data unveiled the intersection of factors across various levels of influence, offering significant insights for understanding the sexual experiences, behaviour, and reproductive health outcomes of PwDs. The sample size was relatively large and as a result the findings can be generalised to the study population in this study’s setting.

However, the study employed cross-sectional design hence, only associations could be established but not causal interpretation of the findings. The qualitative phase however, explored the possible reasons for some of the behaviours considered in the study. All the indicators of sexual behaviours and reproductive health outcomes such as STIs, sexual violence and pregnancy termination were self-reported. As a result, self-reported bias and recall biases cannot be overruled coupled with the sensitive nature of sexual behaviour and abortion. It is therefore possible that some of these indicators are either underreported or overreported. Evidence suggest that PwDs tend to underreport sexual violence due to the stigma attached to it [21, 90]. This fear of relationship breakdown and further stigmatisation discourages them from reporting such cases [21, 76, 90]. However, the respondents were assured of confidentiality and the need to provide accurate responses which made it possible for some of the sexual violence survivors to share their ordeals for the first time. Participants from the quantitative phase who showed interest and consented to participate in the qualitative phase were those who took part, which could contribute to selection bias. The study only included two categories of PwDs–physical and visual impairments. Self-rated SRH was measured with a single item question asking respondents to provide an overall assessment of their SRH health. However, it is widely used in surveys to gauge the health status of populations [99, 103].

## Conclusions

The study highlights that many adults with disabilities have ever had sex and face diverse SRH outcomes, including STIs, unintended pregnancies, and abuse. Factors such as education, gender, ethnicity, income, and access to healthcare influence their perceptions of sexual and reproductive health. Addressing these issues requires coordinated efforts across society, focusing on prevention and management. Further studies could explore a) stakeholder’s perceptions on the strategies to improve the sexual and reproductive health outcomes among PwDs, b) enablers and barriers to help seeking for violence survivors, c) nationwide comparative study on the self-rated SRH of PwDs and people without disability, d) experiences of PwDs seeking abortion and post abortion services.

### Supplementary Information

Supplementary Material 1.

## Data Availability

The dataset is available upon reasonable request from the corresponding authors (A.S and T.I.E).

## Abbreviations

aOR: Adjusted odds Ratio
CI: Confidence Interval
COREQ: Consolidated criteria for reporting qualitative research
GHS: Ghana Health Service
GSS: Ghana Statistical Service
IV: Interpersonal Violence
JCU: James Cook University
KATH: Komfo Anokye Teaching Hospital
NHIS: National Health Insurance Scheme
PwDs: Persons with disabilities
RAs: Research Assistants
SRH: Sexual and reproductive health
UN: United Nations
HPs: Healthcare providers
WHO: World Health Organization
