# Supplementary material for: Sexual lives and reproductive health outcomes among persons with disabilities: a mixed-methods study in two districts of Ghana
Source: Reprod Health. 2024 May 23;21:69. doi: 10.1186/s12978-024-01810-4 (PMC11112844; doi:10.1186/s12978-024-01810-4)
Supplement: Supplementary file 1 — Supplementary Material 1. [file 12978_2024_1810_MOESM1_ESM.pdf]

QUESTIONNAIRE FOR PERSONS WITH DISABILITIES		
**PLEASE READ AND ANSWER THE FOLLOWING QUESTIONS CAREFULLY. YOUR CANDID RESPONSES ARE NEEDED TO GUARANTEE THE RELIABILITY OF THIS STUDY. PLEASE MAKE SURE YOU ANSWER ALL THE QUESTIONS THAT ARE APPLICABLE TO YOU BY CIRCLING THE APPROPRIATE RESPONSE (S).**		
**DATE……………...ENUMERATOR'S INITIALS………..INTERVIEW START………END……………**		
**SECTION 1: CLIENTS CHARACTERISTICS**		
1.1. Sex of respondent	1. Male<br>2. Female	Skip
1.2. Age in completed years	…………………………	
1.3. What is the highest level of education you completed?	1. No formal education<br>2. Primary<br>3. JHS<br>4. SHS/Technical<br>5. Tertiary	
1.4. What is your religion?	1. No religion<br>2. Christianity<br>3. Traditional<br>4. Islamic<br>5. Other specify ……………….	
1.5. What is your current marital status?	1. Never married<br>2. Married<br>3. Cohabiting<br>4. Separated<br>5. Divorced<br>6. Widowed	
1.6. What is your ethnicity	1. Akan<br>2. Ewe<br>3. Ga-Adamgbe<br>4. Mole Dangbani<br>5. Guan<br>6. Gurma<br>7. Other specify…………	
1.7. What is your occupation?	1. Not working/unemployed<br>2. Government employee<br>3. Farming<br>4. Artisan<br>5. Trading<br>6. Other specify……………	
1.8. What is your household size?	Number……………..	
1.9. Who mainly support you in the household?	1. Partner<br>2. Child<br>3. Mother/Father<br>4. Other……….	
1.10. How many children do you have?	Number……………..	
1.11. What is your monthly income?	…………………………..	

1.12. Type of disability	1. Physical<br>2. Visual	
1.13. What is the severity of your disability	1. Mild<br>2. Moderate<br>3. Severe<br>4. Very severe	
1.14. What is the duration of your disability? (in years)	1. Since birth<br>2. …………(years)	
1.15. Have you subscribed to the National Health Insurance Scheme (NHIS)?	1. Yes<br>2. No	
1.16. How long does it take you to travel from your place of residence/house to the nearest health facility?	………. Minutes …..Hours	

**SECTION 2: KNOWLEDGE AND SOURCES OF INFORMATION ABOUT SEXUAL AND REPRODUCTIVE HEALTH SERVICES AND CARE**		
2.1. Have you ever heard about Sexual and Reproductive Health (SRH)?	1. Yes<br>2. No	
2.2. What is/are the importance of accessing SRH services? **(CIRCLE ALL THAT APPLY)**	1. Education on STIs<br>2. Education on personal hygiene<br>3. Getting formation on SRH<br>4. Getting contraceptives<br>5. Helps improve reproductive health<br>6. Other specify…..	
2.3. Have you ever accessed SRH information ?	1. Yes<br>2. No	
2.4. If Yes to (Q. 2.3), from which sources did you access SRH information from? **(CIRCLE ALL THAT APPLY)**	1. Friends and relatives<br>2. Partner<br>3. Health workers<br>4. School<br>5. Radio<br>6. Television<br>7. Information centres<br>8. NGOs<br>9. Churches and mosques<br>10. Disability association<br>11. Other specify ……	
2.5. What is/are the content of the SRH information you accessed? **(CIRCLE ALL THAT APPLY)**	1. Safe sex<br>2. Use of contraception<br>3. Unsafe abortion<br>4. Personal hygiene<br>5. Other specify…………….	
2.6. What prevent you from getting access to SRH information? **(CIRCLE ALL THAT APPLY)**	1. Transportation barrier<br>2. Religious and cultural beliefs<br>3. Partner disapproval<br>4. Inadequate social/community support<br>5. Other specify…….	
2.7. Have you ever heard of Sexually Transmitted Infections (STIs)?	1. Yes<br>2. No	
2.8. Can people take a simple test to find out whether they have an STI?	1. Yes<br>2. No	

2.9. Have you ever heard of HIV and AIDS?	1. Yes<br>2. No	
2.10. Apart from HIV and AIDS, there are other infections that people can get by having sexual intercourse. Have you heard of any of these infections?	1. Yes<br>2. No	
2.11. What are the signs/symptoms of these Sexually transmitted infections? (**CIRCLE EACH MENTIONED)**	1. Discharge from penis/vagina<br>2. Pain during urination<br>3. Ulcers/sores in genital area<br>4. Don't Know any signs<br>5. Other specify……………	
2.12. Do you know of any methods which people can adopt to prevent STIs?	1. Condom use<br>2. Faithfulness to partner<br>3. Abstinence<br>4. Other specify………..	
2.13. Are there methods a woman can adopt to prevent pregancy?	1. Yes<br>2. No	

**SECTION 3: SEXUAL BEHAVIOUR OF PERSONS WITH DISABILITIES (PWDS)**		
3.1. Do you/have you ever had a girl/boyfriend/sexual partner? **(CHECK WITH CURRENT MARITAL STATUS)**	1. Yes<br>2. No	
3.2. How many girls/boyfriends/partners have you had in the last 12 months?	Number……………	
3.3. Have you ever had sex?	1. Yes<br>2. No	
3.4. How old were you at the time you had your first sexual intercourse?	Age (years)……………	
3.5. Did you use any contraception during your first sex?	1. Yes<br>2. No	
3.6. Did you have your first sexual intercourse because you wanted it or you were forced?	1. Yes, I wanted to<br>2. No, I was forced/lured to	
3.7. Have you ever tested for HIV/AIDS?	1. Yes<br>2. No	

**SECTION 4: UPTAKE OF SRH SERVICES AND INTERVENTIONS**		
4.1. Have you ever heard of any health policy/ intervention?	1. Yes<br>2. No	
4.2. If Yes, from where?	1. Radio<br>2. Television<br>3. Friends<br>4. Disability group<br>5. Health workers<br>6. NGOS<br>7. Other specify…………….	
4.3. Name of health policy/intervention	……………………..	
4.4. Which of the Health policies interventions are solely for PWDs?	…………………….	
4.5. Have you ever visited a health facility or doctor of any kind to receive services or information on contraception, pregnancy, abortion or STIs when you had the need to?	1. Yes<br>2. No	
4.6. If No why? (**CIRCLE ALL THAT APPLY**)	1. Cost of health service<br>2. Physical barriers to the facility<br>3. Discrimination by professionals	

	4. Distance to service point<br>5. Lack of medical equipment<br>6. Adapted for my usage<br>7. Lack of communication access<br>8. Religious belief<br>9. Cultural belief<br>10. Other specify:........................	
4.7. Thinking about your last visit, did you go to a government clinic, health centre or hospital or a private doctor or clinic?	1. Govt. hospital/health centre/clinic<br>2. Private doctor/nurse/clinic<br>3. Other specify ……	
4.8. Please tell me the name of the facility you have ever accessed SRH services?	…………………………………	
4.9. When you last accessed SRH services, what was your reason?	1. Contraception<br>2. STI treatment<br>3. Gynaecological exam<br>4. Pregnancy test<br>5. Pregnancy Termination<br>6. Post-natal care<br>7. HIV test<br>8. ANC<br>9. Other specify………	
4.10. What enabled you to access the service? **(CIRCLE ALL THAT APPLY)**	1. Preferential Treatment<br>2. Support for Transport/from Caregivers<br>3. Health providers Attitudes<br>4. National Health Insurance<br>5. Other specify…..	
4.11. Did you get well after the treatment?	1. Yes<br>2. No	
4.12. Were you satisfied with the reception of the person who attended to you?	1. Yes<br>2. No	
4.13. Did you face any discrimination when you accessed the SRH services?	1. Yes<br>2. No	
4.14. Did you face any challenge(s) when you started accessing the service?	1. Yes<br>2. No	
4.15. If Yes to (Q.4.12), what challenge(s) did you face? **(CIRCLE ALL THAT APPLY)**	1. Cost of health service<br>2. Physical barriers<br>3. Discrimination by professionals<br>4. Lack of medical equipment<br>5. Adapted for my usage<br>6. Problem of communication<br>7. Other specify ………	
4.16. Making reference to the last time you accessed SRH services, how will you rate the services you were offered on the scale of "very satisfied=1 to very disasatified=4?"	1. Very satisfied<br>2. satisfied<br>3. Dissatisfied<br>4. Very dissatisfied	

SECTION 5: IMPLEMENTATION, UPTAKE AND EFFECTIVENESS OF POLICIES AND PROGRAMMES AIMED AT IMPROVING SRH OF PWDS		
**Indicate whether you agree or disagree that the following factors influence the implementation of SRH services among PWDs in Ghana**		
5.1. Sexual violence against PWDs is one of the factors that influence the implementation of SRH interventions for PWDs.	1. Strongly agree<br>2. Agree<br>3. Disagree<br>4. Strongly disagree	
5.2. Physical violence against PWDs is one of the factors that influence the implementation of SRH interventions for PWDs.	1. Strongly agree<br>2. Agree<br>3. Disagree<br>4. Strongly disagree	
5.3. Emotional violence against PWDs is one of the factors that influence the implementation of SRH interventions for PWDs	1. Strongly agree<br>2. Agree<br>3. Disagree<br>4. Strongly disagree	
5.4. Coercive sex against PWDs is one of the factors that influence the implementation of SRH interventions for PWDs	1. Strongly agree<br>2. Agree<br>3. Disagree<br>4. Strongly disagree	
5.5. Multiple sexual partners among PWDs is one of the factors that influence the implementation of SRH interventions for PWDs	1. Strongly agree<br>2. Agree<br>3. Disagree<br>4. Strongly disagree	
5.6. Low contraceptive among PWDs is one of the factors that influence the implementation of SRH interventions for PWDs	1. Strongly agree<br>2. Agree<br>3. Disagree<br>4. Strongly disagree	
5.7. Lack of affordable and adequate contraceptive use among PWDs is one of the factors that influence the implementation of SRH interventions for PWDs	1. Strongly agree<br>2. Agree<br>3. Disagree<br>4. Strongly disagree	
5.8. Inconsistent and incorrect condom use among PWDs is one of the factors that influence the implementation of SRH interventions for PWDs	1. Strongly agree<br>2. Agree<br>3. Disagree<br>4. Strongly disagree	
5.9. High poverty among PWDs is one of the factors that influence the implementation of SRH interventions for PWDs	1. Strongly agree<br>2. Agree<br>3. Disagree<br>4. Strongly disagree	
5.10. High illiteracy among PWDs is one of the factors that influence the implementation of SRH interventions for PWDs	1. Strongly agree<br>2. Agree<br>3. Disagree<br>4. Strongly disagree	

5.11. Unemployment among PWDs is one of the factors that influence the implementation of SRH interventions for PWDs	1. Strongly agree<br>2. Agree<br>3. Disagree<br>4. Strongly disagree	
5.12. Please indicate whether these intervention(s) have been implemented to improve SRH of PWDs (**CIRCLE ALL THAT APPLY).**	1. Sexual and reproductive health Information provision<br>2. STIs Testing and Treatment<br>3. Provision of general reproductive health services<br>4. Comprehensive sexuality education<br>5. Youth empowerment<br>6. Training of healthcare providers<br>7. Education of community members	
PLEASE RATE THE EFFECTIVENESS OF THE FOLLOWING PROGRAMS/INTERVENTIONS AIMED AT IMPROVING THE SRH OF PWDS		
5.13. Information provision by the government, health officials and NGOS on SRH	1. Very effective<br>2. Effective<br>3. Ineffective<br>4. Very ineffective	
5.14. Provision and delivery of reproductive health services	1. Very effective<br>2. Effective<br>3. Ineffective<br>4. Very ineffective	
5.15. Comprehensive sexuality education	1. Very effective<br>2. Effective<br>3. Ineffective<br>4. Very ineffective	
5.16. Youth empowerment on SRH issues	1. Very effective<br>2. Effective<br>3. Ineffective<br>4. Very ineffective	
5.17. Training of healthcare providers on how to render SRH services to PWDs	1. Very effective<br>2. Effective<br>3. Ineffective<br>4. Very ineffective	
5.18. Education of community members on SRH issues in relation to PWDs	1. Very effective<br>2. Effective<br>3. Ineffective<br>4. Very ineffective	
5.19. STIs testing and Treatment for PWDs	1. Very effective<br>2. Effective<br>3. Ineffective<br>4. Very ineffective	
5.20. Overall, how effectiveness has been the programs/interventions aimed at improving the SRH of PWDs	1. Very effective<br>2. Effective	

	3. Ineffective<br>4. Very ineffective	

SECTION 6: SEXUAL AND REPRODUCTIVE HEALTH OUTCOMES		
6.1. **MALES:** Have you ever impregnanted your girlfriend? Did girlfriend/partner ever become pregnant by you?<br>**FEMALES:** Did you ever become pregnant by boyfriend/partner?	1. Yes<br>2. No	
6.2. **WOMEN**<br>If Yes to (Q. 6.1) during the pregnancy did you seek Antenatal Care	1. Yes……..(times)<br>2. No	
6.3. Where did you deliver/give birth?	1. Hospital<br>2. Home<br>3. Other specify………	
6.4. After delivery, did you go for postnatal checks?	1. Yes<br>2. No	
6.5. Some women lose their pregnancy spontaneously, that is they have a miscarriage. Have you ever had a miscarriage?	1. Yes…….(times/no)<br>2. No	
6.6. Have you ever experienced any still birth?	1. Yes…….(times/no)<br>2. No	
6.7. Have you ever terminated a pregnancy before?	1. Yes…..<br>2. No	
6.8. If YES to (Q. 6.7), what did you do?	1. Took concoction<br>2. Went to the hospital<br>3. Other specify …….	
6.9. **Physical violence:**<br>Has someone push you, shake you, or throw something at you; slap you; twist your arm or pull your hair; punch you with his fist or with something that could hurt you; kick you, drag you, or beat you up; try to choke you or burn you on purpose; or threaten or attack you with a knife, gun, or any other weapon?	1. Yes<br>2. No	
6.10. Who did this to you?	1. Partner<br>2. Other family member<br>3. Immediate care giver<br>4. Other specify……..	
6.11. **Sexual violence:**<br>Has someone physically force you to have sexual intercourse with him/her even when you did not want to, physically force you to perform any other sexual acts you did not want to, or force you with threats or in any other way to perform sexual acts you did not want to.	1. Yes<br>2. No	
6.12. If Yes to Q6.11, Who did this to you?	1. Partner<br>2. Other family member<br>3. Immediate care giver<br>4. Other specify……	
6.13. **Emotional violence:**<br>Has someone said or did something to humiliate you in front of others, threaten to hurt or harm you or someone close to you, or insult you or make you feel bad about yourself?	1. Yes<br>2. No	
6.14. If Yes to Q6.13 above, who did this to you?	1. Partner<br>2. Other family member<br>3. Immediate care giver<br>4. Other specify…..	
6.15. During the last 12 months, have you had a disease which you got through sexual intercourse?	1. Yes<br>2. No	
**WOMEN:**	1. Yes<br>2. No	

6.16. Sometimes women experience a bad-smelling abnormal genital discharge. During the last 12 months, have you had a bad-smelling abnormal genital discharge?<br>**MEN:**<br>Sometimes men experience an abnormal discharge from their penis. During the last 12 months, have you had an abnormal discharge from your penis?		
**WOMEN:**<br>6.17. Sometimes women have a genital sore or ulcer. During the last 12 months, have you had a genital sore or ulcer?<br>**MEN:**<br>Sometimes men have a sore or ulcer near their penis. During the last 12 months, have you had a sore or ulcer near your penis?	1. Yes<br>2. No	
6.18. If Yes to (Q. 6.17), what type of STI(s) did you contract?	1. Gonorrhoea<br>2. Syphilis<br>3. Chlamydia<br>4. HIV and AIDS<br>5. Other specify…. …	
6.19. IF Yes to (Q. 6.17), how many times?	…………….	
6.20. On the last occasion did you seek medical treatment?	1. Yes<br>2. No	
6.21. Where did you seek medical treatment?	1. Shop<br>2. Pharmacy<br>3. Govt. hospital/health centre/clinic<br>4. Private doctor/nurse/clinic<br>5. Other specify….	
6.22. Did your sexual partner (any of your partners) also obtain medical treatment?	Yes<br>No	
6.23. If No to (Q. 6.22) why?	………………..	
6.24. The sex I have had was always as safe as I wanted it to be because of the SRH interventions I have beneifited from	1. Yes<br>2. No	
6.25. Sexual autonomy: "I have always been able to refuse sexual practice/s I don't want" because of the SRH interventions I have beneifited from	1. Yes<br>2. No	
6.26. Overall, how will you rate your sexual and reproductive health status?	1. Very good<br>2. Good<br>3. Bad<br>4. Very bad	
6.27. Overall, how satisfied are you with your life nowadays?	1. Very satisfied<br>2. Satisfied<br>3. Dissatisfied<br>4. Very dissatisfied	
6.28. Have you taken the COVID-19 vaccine?	1. Yes<br>2. No	
6.29. Are you willing to take part in an indepth interview?	1. Yes<br>2. No	
6.30. If yes to 29, please provide contact details/phone number.	…………………………	
